# Inhibition of stationary phase respiration impairs persister formation in *E. coli*

**DOI:** 10.1038/ncomms8983

**Published:** 2015-08-06

**Authors:** Mehmet A. Orman, Mark P. Brynildsen

**Affiliations:** 1 Department of Chemical and Biological Engineering, Princeton University, Princeton, New Jersy 08544, USA

## Abstract

Bacterial persisters are rare phenotypic variants that temporarily tolerate high antibiotic concentrations. Persisters have been hypothesized to underlie the recalcitrance of biofilm infections, and strategies to eliminate these cells have the potential to improve treatment outcomes for many hospital-treated infections. Here we investigate the role of stationary phase metabolism in generation of type I persisters in *Escherichia coli*, which are those that are formed by passage through stationary phase. We find that persisters are unlikely to derive from bacteria with low redox activity, and that inhibition of respiration during stationary phase reduces persister levels by up to ∼1,000-fold. Loss of stationary phase respiratory activity prevents digestion of endogenous proteins and RNA, which yields bacteria that are more capable of translation, replication and concomitantly cell death when exposed to antibiotics. These findings establish bacterial respiration as a prime target for reducing the number of persisters formed in nutrient-depleted, non-growing populations.

## Abstract (web summary)

A few bacterial cells within a genetically homogeneous population can become ‘persisters’, or temporarily tolerant to antibiotics. Here Orman and Brynildsen show that development of persisters among growth-arrested *E. coli* cells can be prevented by inhibiting bacterial respiration.

## Graphical abstract

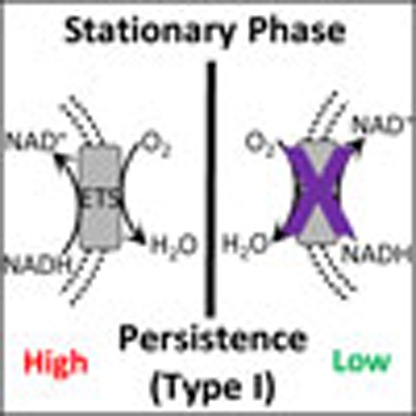

Persisters comprise a small fraction of a bacterial population that is temporarily tolerant to antibiotics even though they are genetically identical to their antibiotic-susceptible kin^1^,^2^. It has been hypothesized that persisters are a major reason underlying the proclivity of biofilm infections to relapse, since they are protected from host immune cells by the physical barrier provided by the biofilm extracellular matrix and protected from antibiotics due to their own tolerances^3^,^4^. Anti-persister therapies promise to provide better treatment outcomes for biofilm infections^3^,^5^,^6^,^7^,^8^, which constitute the majority of infections treated in US hospitals^9^, yet strategies to eliminate persisters remain sparse due to limited knowledge of the persister phenotype^1^,^4^,^10^. This knowledge gap continues to exist because persisters are transient and phenotypically similar to the more abundant ‘viable but non-culturable cells’, thereby impeding efforts to produce high-purity persister samples from native populations^11^,^12^,^13^. Despite this complication, it is known that persisters are more abundant in slowly or growth-arrested populations^2^,^14^,^15^,^16^,^17^,^18^,^19^, though they remain rare even in non-growing cultures^7^,^20^,^21^. What determines whether one non-growing cell succumbs to antibiotics or gives rise to a persister remains ill-defined, as elegantly portrayed in the recent studies by Maisonneuve *et al*.^16^ where not all bacteria growth arrested by stochastic ppGpp accumulation gave rise to persisters.

Here we investigate whether the metabolic activity of a growth-arrested cell affects its likelihood of being a persister. Specifically, we investigate type I persisters, which are those that are generated by passage through stationary phase and enumerated by exposure of stationary phase cells to antibiotics in fresh media^2^,^19^,^22^,^23^. Previously, we used a fluorescent measure of metabolic activity (Redox Sensor Green (RSG) dye), fluorescence-activated cell sorting (FACS), and persistence assays to provide the first direct measurement of persister metabolism in exponentially growing cultures^17^. Here we investigate stationary phase cultures, as a model non-growing system, and measure the abundance of type I persisters. We find that persisters are largely absent from the subpopulation with the lowest level of redox activity. We dissect this phenotype with genetic, biochemical, and flow-cytometric techniques and discover that inhibition of respiration within this nutrient-depleted phase is an effective means to prevent persister formation. Further, from measurements of cell size, protein degradation and RNA integrity, we demonstrate that self-digestion is associated with the formation of persisters in stationary phase. These results identify respiration as a potential source of anti-persister strategies that is distinct from previously identified targets^5^,^7^,^8^,^16^,^24^,^25^.

## Results

### Type I persisters are rare in cells with low redox activity

RSG is a fluorogenic redox indicator that yields green fluorescence when reduced by bacterial reductases^26^,^27^,^28^. This dye does not affect the culturability and persistence of *Escherichia coli* cells, and staining of bacterial cells is significantly attenuated by metabolic inhibitors^17^. Using FACS, RSG dye and antibiotic-tolerance assays we had found that in exponentially growing cultures persisters were significantly more abundant in the least redox active subpopulation^17^. Since metabolism plays a significant and increasingly appreciated role in persistence^1^,^4^,^7^,^11^,^29^,^30^,^31^, we tested whether stationary phase metabolic activity played a decisive role in defining those bacteria that would become type I persisters. To verify that RSG staining of stationary cells reflected metabolic activity, we treated stationary phase cultures with ethanol and observed that these dead, metabolically inactive cells could not be stained (Supplementary Fig. 1). In addition, we added glucose to stationary phase cultures to boost their metabolism and observed that RSG staining was significantly increased over the untreated control (Supplementary Fig. 1).

**Figure 1:**
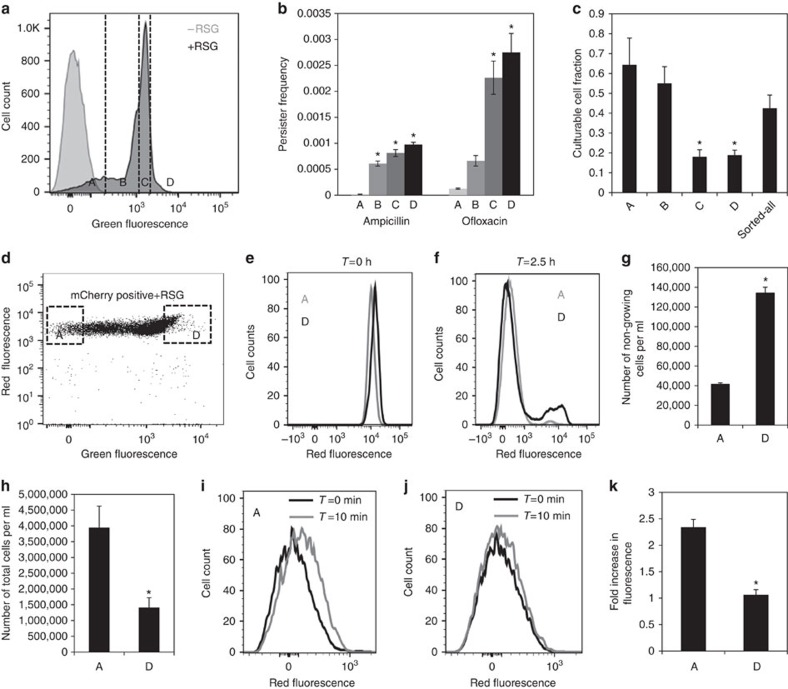
Stationary phase metabolic activity and persistence. (**a**) Overnight cultures (24 h) were stained with RSG for 1 h, and then segregated with FACS into four quantiles, A, B, C and D, comprising 10, 40, 40 and 10% of total population. (**b**) Segregated cells diluted in fresh LB were treated with 200 μg ml^−1^ ampicillin or 5 μg ml^−1^ ofloxacin for 5 h to enumerate the persister levels. These treatment conditions produced biphasic killing as depicted in Supplementary Figure 2A. (**c**) Shortly after sorting, segregated samples with known number of cells were plated on LB agar to count the c.f.u. Culturable cell fraction is the ratio of the number of c.f.u. to total number of cells as determined by FACS. (**d**) Overnight cultures with MO001 cells where mCherry protein was expressed with 1 mM IPTG were stained with RSG, and then A and D subpopulations were segregated with FACS. Unstained control of mCherry-positive cells is provided in Supplementary Figure 7A. (**e,f**) Segregated subpopulations (A:grey, D:black) were diluted in fresh LB without IPTG and then cultured for 2.5 h at 37 °C with shaking. At *t*=0 h, and 2.5 h, samples were analysed with flow cytometry to quantify the mCherry protein at single cell level. (**g**,**h**) The number of total cells and non-growing cells in these cultures at *t*=2.5 h were enumerated with counting beads. Non-growing cells retained their mCherry levels, whereas growing cells had reduced mCherry levels due to cell division. (**i**,**k**) Overnight cultures with *E. coli* cells carrying pQE-80L*mCherry* (without IPTG) were stained with RSG, and then A and D subpopulations were segregated with FACS. Cells were diluted in fresh LB with 1 mM IPTG, and mCherry was measured with flow cytometry at *t*=0 (black), and 10 min (grey). The fold changes in fluorescence for both A and D subpopulations after 10 min of culturing are shown in **k**. ‘*’ signifies significant differences for comparisons to subpopulation A (*P*-value<0.05, *t*-test). At least three biological replicates were performed for each experimental condition. Each data point was denoted by mean value±s.e.

To assay the dependence of persistence on stationary phase metabolism, we stained 24-h overnight cultures of *E. coli* with RSG, segregated the population with FACS into four subpopulations (A, B, C, D) comprising 10, 40, 40 and 10% of the population as depicted in Fig. 1a, and assayed for type I persistence by treating the sorted cells with ampicillin or ofloxacin in fresh media and measuring the level of colony-forming units per ml as a function of time. Biphasic killing verified that 5 h of antibiotic treatment was sufficient to quantify persister levels and sorting and segregation controls indicated that flow through the sorter and segregation into quantiles did not alter persister levels (Supplementary Fig. 2). Persistence to aminoglycosides was also measured, but the levels were at or below the level of detection of the assay (∼10 c.f.u. ml^−1^) (Supplementary Fig. 3), and therefore, not further investigated here. However, we note that such differences in type I persister levels between aminoglycosides and other drug classes have been observed previously^12^. The ampicillin and ofloxacin persister frequencies in A, B, C and D demonstrated that stationary phase cells with comparatively lower redox activity (region A) are >20-fold less likely to give rise to type I persisters than stationary phase cells with higher redox activity (region D; Fig. 1b). To elucidate whether cells in A or D regions acquire mutations that might lead to changes in persistence, cells from A and D were inoculated into fresh media and grown until stationary phase. When persister assays were performed on these cultures, both ampicillin and ofloxacin persister levels in the A- and D-derived cultures were identical to the parental strain (Supplementary Fig. 4), indicating that neither the A nor D subpopulation acquired mutations.

### Loss of culturability correlates with high redox activity

We sought to determine whether the decrease in persister levels observed in region A was due to culturability differences between the subpopulations. Since the persister frequencies in Fig. 1b were calculated based on the total number of cells sorted in each quantile, the lower persister frequency in region A could have derived from a lower culturability of cells in region A compared to other quantiles. However, the culturability of cells in region A was found to be higher than that in region D (Fig. 1c). We note that the dependence of persistence on RSG staining is a graded response (Fig. 1a–c) and we chose to focus on quantiles A and D because they comprise the extremes. Interestingly, if persister frequencies are calculated based on culturable cells, rather than total cells, this increases the difference in persister frequency observed between regions A and D from 20-fold to >70-fold. Due to the reduction in culturability of cells that stained the most with RSG, we assessed whether the phenotype observed could be accounted for by differences in membrane permeability, rather than metabolic activity. Therefore, we stained stationary phase cells with propidium iodide (PI), which can only penetrate cells with compromised membranes and segregated the population into four subpopulations (Supplementary Fig. 5A,B). When persistence and culturability in these subpopulations were measured, persister frequencies and culturable cell fractions were equivalent across all gates (Supplementary Fig. 5C,D), showing that the phenotype could not be explained by membrane permeability as measured by PI. To determine whether increased efflux of RSG may underlie poor staining of the A subpopulation, we assayed RSG staining of a strain devoid of TolC, which is an essential component of the major efflux pumps in *E. coli*^32^. Interestingly, Δ*tolC* failed to increase RSG staining over the wild-type control (Supplementary Fig. 6), which suggested that increased activity of efflux pumps were not responsible for poor staining of the A subpopulation. Collectively, these data provide evidence that increased metabolic activity within stationary phase led to loss of culturability and an increase in the formation of type I persisters.

### High redox activity yields more non-growing cells

Given these results and previous work that has found significantly higher persister levels in subpopulations of cells that fail to rapidly resume growth on exposure to fresh nutrients^2^,^13^,^15^,^17^,^19^, we hypothesized that the A subpopulation might have fewer non-growing cells after inoculation into fresh media than the more metabolically active subpopulations. Therefore, we tracked cell division after resuspension of A and D subpopulations in fresh media by dilution of a fluorescent protein (mCherry) using an *E. coli* strain (MO001)^17^. To monitor cell division, *mCherry* expression was induced during the overnight growth, which did not alter the persister levels (Supplementary Fig. 7), the culture was stained with RSG (Fig. 1d), A and D subpopulations were segregated with FACS (Fig. 1d), and inoculated into fresh media without inducer. The dilution of mCherry by cell division was monitored using flow cytometry. As depicted in Fig. 1e,f, all cells started with high red fluorescence (*t*=0 h) and as cells divided the red fluorescence of the populations declined, except for small subpopulations in both A and D (non-growing cells) whose fluorescence remained constant due to lack of division (*t*=2.5 h). When the growing and non-growing cells were counted at *t*=2.5 h, we observed that subpopulation A had significantly more growing and significantly fewer non-growing cells compared to the D subpopulation (Fig. 1g,h). Even though the initial number of cells at *t*=0 h was the same in both cultures (∼200,000 cells per ml), the total number of cells in the A subpopulation was 2.8-fold higher than the D subpopulation at *t*=2.5 h (Fig. 1h). At 2.5 h, the number of non-growing cells in the A-derived culture was 41,400±1,500 ml^−1^ and 136,600±6,500 ml^−1^ in the D-derived culture. This implied that 158,600±1,500 cells from the A subpopulation sample and 63,400±6,500 cells from the D subpopulation sample had resumed growth, which indicated that 2.59±0.29-fold more cells from the A subpopulation resumed growth compared with cells from the D subpopulation. With the assumptions that growing cells from A- and D-derived cultures grew at comparable rates and initiated growth similarly, this difference would manifest as a 2.59±0.29-fold difference in cell density once within the exponential phase of growth. Since this ratio was not significantly different than that measured after 2.5 h of growth (2.8-fold), the data suggest that a higher proportion of the A subpopulation resumed growth compared to the D subpopulation. Similar to wild-type (WT) results, both ampicillin and ofloxacin type I persister levels in the A subpopulation of MO001 were significantly reduced (Supplementary Fig. 7B). In our previous study, after inoculating stationary phase MO001 cells in fresh media and growing the cells for 2.5 h, we demonstrated that the vast majority of persisters in *E. coli* cultures arose from the non-growing subpopulation^17^. Given the identical growth conditions used between that study and this one, we can conclude that the majority of persisters from each region (A or D) originated from cells that do not rapidly resume growth on introduction into fresh media, and the observation of far fewer persisters in region A compared with region D is consistent with the significantly reduced abundance of non-growing cells that arise from this subpopulation.

### Redox activity inversely correlates with protein synthesis

To determine whether the A subpopulation had a heightened ability to generate protein, which would be reflective of more competent transcription and translation machinery, we measured the protein synthesis capabilities of the A and D subpopulations ten minutes after inoculation into fresh media. We performed this assay shortly after inoculation (10 min) to measure transcriptional and translational capabilities prior to cell proliferation. For this assay, we used WT *E. coli* carrying a pQE-80L plasmid expressing *mCherry* from an isopropyl-b-D-thiogalactoside (IPTG) inducible promoter. After staining with RSG, the subpopulations were segregated with FACS and inoculated in fresh media with IPTG. Ten minutes after inoculation, mCherry was detectable in the A subpopulation whereas it was undetectable in the D subpopulation (Fig. 1i–k). Collectively, these data (Fig. 1) indicate that lower redox activity in stationary phase leads to cells that are primed for transcription and translation on exposure to fresh nutrients, and this physiology largely prevents the formation of type I persisters.

### Respiratory inhibition prevents type I persister formation

**Figure 2:**
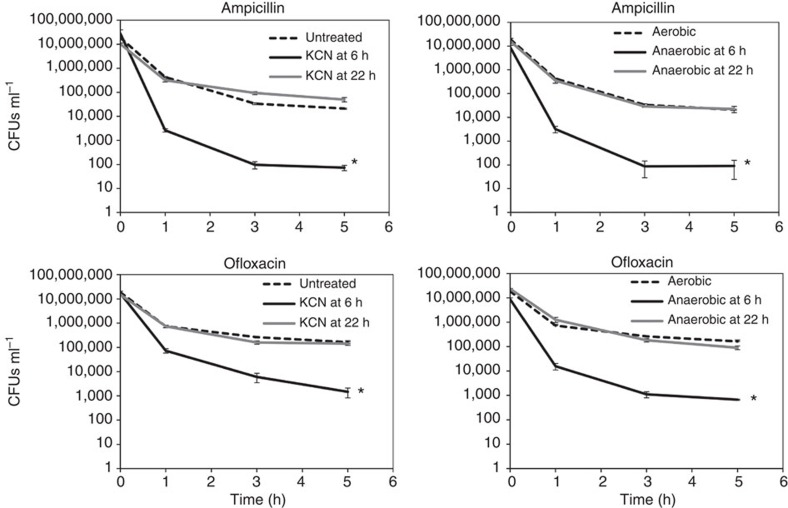
Impact of inhibition of stationary phase respiration on type I persister levels. Cultures at *t*=6 h or 22 h were treated with 1 mM KCN or transferred to an anaerobic chamber. At *t*=24 h, cultures were washed to remove the chemical inhibitors and diluted (100-fold) in fresh LB and treated with ampicillin or ofloxacin. c.f.u. ml^−1^ levels were monitored for 5 h during the treatments. Note that for controls, the cells were treated with solvent, H_2_O. ‘*’ signifies significant differences for comparisons with the control group, which was untreated, aerobic cultures here (*P*-value<0.05, *t*-test). At least three biological replicates were performed for each experimental condition. Each data point was denoted by mean value±s.e.

During stationary phase, type I persister levels increase by orders of magnitude^12^ as evidenced in Supplementary Fig. 8, and the data in Fig. 1 suggested that stationary phase redox activity was critical for such formation. We sought to identify a specific metabolic process that was associated with these observations, which was unclear due to the non-specific capacity of RSG to be reduced by different bacterial enzymes^26^,^27^,^28^. Since respiration is a major redox process in aerobically grown cultures, we hypothesized that inhibition of stationary phase respiration may be an effective means to prohibit type I persister formation. We considered two scenarios, the first was that respiratory activity during the time period associated with RSG staining was critical to persister formation, or alternatively, respiration throughout stationary phase was required. To test the first scenario, we inhibited respiration at late stationary phase (*t*=22 h) (Supplementary Fig. 8A) with 1 mM potassium cyanide (KCN) or transferred cultures to an anaerobic chamber. At *t*=24 h, cells were washed to remove KCN, diluted into fresh media, and treated with ampicillin or ofloxacin. It was observed that inhibition of respiration at late stationary phase did not alter the persister levels (Fig. 2), which demonstrated that cellular respiratory activity at late stationary phase did not contribute to type I persister formation. To determine whether inhibition of respiration throughout stationary phase would inhibit persister formation, cells were treated with KCN or were transferred to anaerobic conditions at early stationary phase (*t*=6 h) (Supplementary Fig. 8A), and persister levels in these cultures were measured at *t*=24 h. We observed that inhibition of respiration throughout stationary phase significantly decreased persister levels (Fig. 2). We also found that KCN significantly reduced persister levels at various concentrations (0.5, 1 and 2 mM; Supplementary Fig. 9). Since the duration of KCN treatment was far greater for the second scenario (18 h) compared with the first (2 h), we treated late stationary phase cells at *t*=22 h with KCN for 18 h, and observed that this did not alter persister levels when compared to untreated controls (Supplementary Fig. 10).

**Figure 3:**
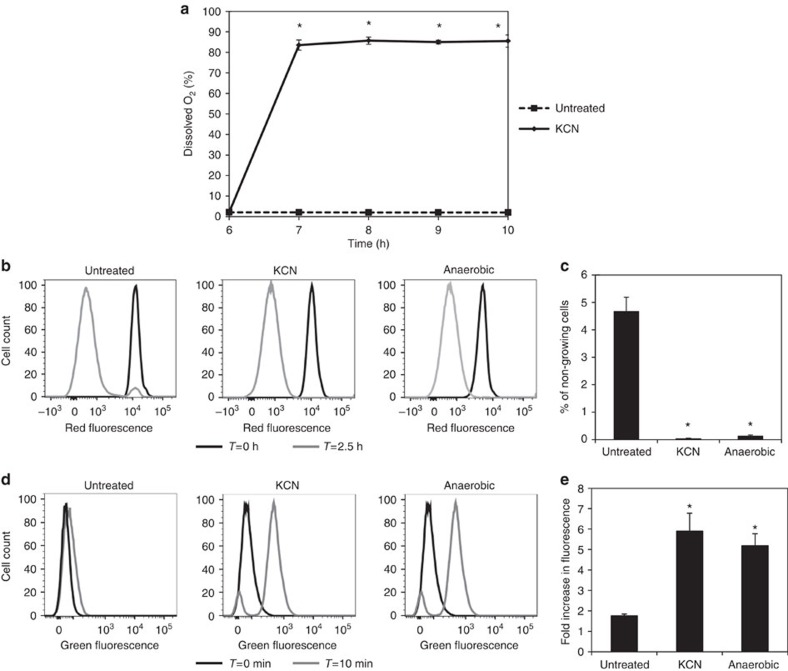
Dissolved oxygen, non-growing cell abundance and protein expression levels following inhibition of stationary phase respiration. (**a**) Percentages of dissolved oxygen concentrations in cell cultures with respect to saturated media were determined after treatment with KCN. (**b**,**c**) Overnight cultures of MO001 cells where mCherry was expressed with 1 mM IPTG were similarly treated with KCN or transferred to an anaerobic chamber at *t*=6 h. At *t*=24 h, cells were washed and diluted in fresh LB, and the non-growing cells were enumerated at *t*=2.5 h with flow cytometry. (**d**,**e**) Overnight cultures with *E. coli* cells carrying pQE-80L*gfp* (without IPTG) were treated with KCN or transferred to an anaerobic chamber at *t*=6 h. At *t*=24 h, cells were washed and diluted in fresh LB with the inducer, and GFP expression was monitored within 10 min with flow cytometry. Note that for controls, the cells were treated with solvent, H_2_O. ‘*’ signifies significant differences for comparisons with the untreated group (*P*-value<0.05, *t*-test). At least three biological replicates were performed for each experimental condition. Each data point was denoted by mean value±s.e.

To confirm that stationary phase respiration was inhibited when cultures were treated with KCN, dissolved oxygen concentrations in cell cultures with and without KCN treatment were measured. As depicted in Fig. 3a, KCN treatment at *t*=6 h significantly impaired O_2_ consumption when compared with untreated cultures. Since protein synthesis and the proportion of cells that remained non-growing after 2.5 h in fresh media were characteristics that differed between the A- and D-subpopulations of RSG-stained WT cultures, we tested whether KCN treatment and transfer to anaerobic conditions reduced persisters in a similar manner to that observed for the A subpopulation. We observed that both KCN and transfer to anaerobic conditions before stationary phase significantly reduced the non-growing subpopulation, and protein synthesis when compared with untreated controls (Fig. 3b–e). When cultures treated with KCN or transferred to anaerobic conditions were compared with untreated or non-transferred controls, the differences in physiology between treated/untreated and transferred/non-transferred closely mirrored those observed between A- and D-subpopulations, which suggested that respiratory inhibition impaired type I persister formation through a mechanism that was similar to that underlying the low frequency of persisters in the A subpopulation of untreated cultures.

To determine whether the reduction in persisters was specific to respiratory inhibition or more generally associated with the growth inhibition that results from such treatments (Supplementary Fig. 11), cells at early stationary phase were treated with rifampicin (RIF) or chloramphenicol (CAM) to inhibit macromolecular synthesis (RNA and protein, respectively). Neither RIF nor CAM at 25, 50 or 100 μg ml^−1^ reduced persister levels (Supplementary Fig. 12), confirming that inhibition of respiration, and not the associated growth inhibition, was responsible for preventing persister formation. Oxygen concentrations in cultures that were treated with RIF and CAM were found to be very similar to untreated cultures (Supplementary Fig. 13C), which demonstrated that neither RIF nor CAM inhibited stationary phase respiration. The non-growing subpopulation, and initial protein synthesis were also measured on inoculation of RIF- or CAM-treated cells into fresh media (Supplementary Fig. 13A,B). However, neither of these treatments reduced the persistence, reduced the non-growing cell population, or improved protein synthesis.

### Deletion of TCA cycle enzymes reduce persister formation

To mechanistically interrogate how inhibition of stationary phase respiration reduced persister formation by up to 1,000-fold (Fig. 2), we sought to determine whether the phenomenon was associated with other perturbations that were previously identified to reduce persister formation. Specifically, ppGpp, Lon, DksA and several genes associated with metabolism, such as *sucB*, *mdh, icdA, acnB, phoU* and *ubiF*, have been identified as mediators of persistence, whose removal from *E. coli* leads to significantly reduced persister levels^12^,^16^,^24^,^25^,^33^,^34^. Under the conditions used here, Δ*relA*Δ*spoT*, Δ*lon*, Δ*dksA*, Δ*sucB*, Δ*mdh*, Δ*acnB*, and Δ*ubiF* all significantly reduced type I persister levels (Supplementary Figs 14A and 15A). However, only Δ*sucB*, Δ*mdh* and Δ*ubiF* exhibited both significantly lower levels of non-growing cells and significantly improved initial protein synthesis on introduction into fresh media (Supplementary Figs 14B,C and 15B,C). In addition, Δ*sucB*, Δ*mdh* and Δ*ubiF* were also the mutants that perturbed stationary phase respiration to the greatest extent (Supplementary Fig. 16). The only other mutant to significantly alter respiration was Δ*icdA*, but the effect was not sustained as it was in Δ*sucB*, Δ*mdh*, and Δ*ubiF*. UbiF is an enzyme involved in biosynthesis of ubiquinone, which is a respiratory electron carrier, SucB is a component of the NADH-producing 2-ketoglutarate dehydrogenase complex, and Mdh encodes malate dehydrogenase, which catalyses the oxidation of malate to oxaloacetate, while generating NADH. Succinate dehydrogenase catalyses an enzymatic step between 2-ketoglutarate dehydrogenase and malate dehydrogenase that also produces reducing power for respiration in the form of ubiquinol (UQH_2_), and deletion of one of its essential components (Δ*sdhC*) similarly yielded lower levels of persisters and non-growing cells, perturbed stationary phase respiration and increased protein synthesis on inoculation into fresh media when compared with WT (Supplementary Figs 15 and 16). These results indicated that removal of enzymes that play important roles in respiration during stationary phase produced similar phenotypes to those observed for cultures whose respiratory activities were inhibited by KCN or transfer to anaerobic conditions. Interestingly, Δ*relA*Δ*spoT*, Δ*lon* and Δ*dksA*, despite having significantly reduced type I persister levels, respired normally on entry into stationary phase, did not reduce the abundance of non-growing cells nor exhibit significantly improved protein synthesis in comparison to WT on exposure to fresh nutrients (Supplementary Figs 14 and 16). Altogether, data from these mutants provides evidence that stationary phase respiratory activity is necessary but not sufficient for type I persister formation in *E. coli*.

### ROS are not involved in persister formation

**Figure 4:**
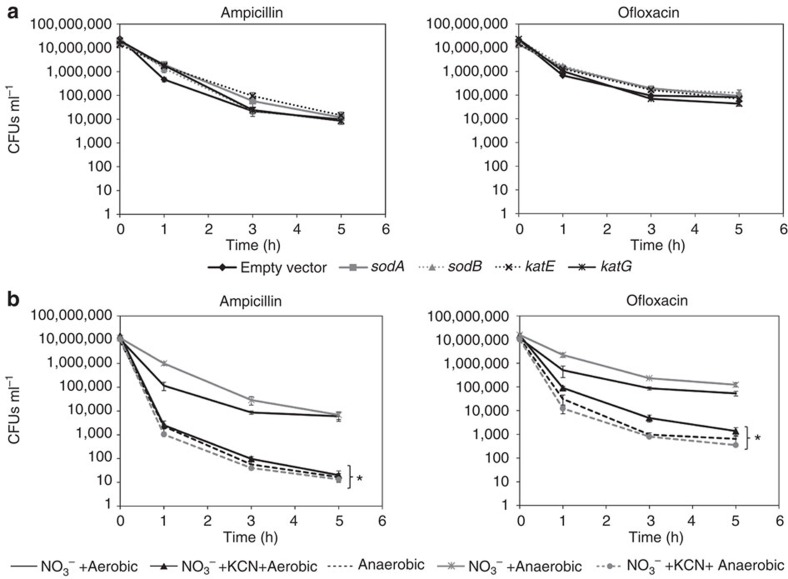
Persister levels in strains overexpressing catalases and superoxide dismutases and cultures respiring anaerobically. (**a**) *sodA*, *sodB*, *katE* and *katG* were overexpressed at *t*=6 h in overnight cultures using pQE-80L plasmid, and at *t*=24 h, cells were washed to remove the inducer and diluted in fresh media for persister assay. (**b**) Wild-type (WT) cell cultures at *t*=6 h were treated with 40 mM NaNO_3_ and/or 1 mM KCN and/or transferred to an anaerobic chamber. At *t*=24 h, cultures were washed to remove the chemicals and diluted (100-fold) in fresh LB and treated with ampicillin or ofloxacin aerobically. c.f.u. levels were monitored for 5 h during the treatments. ‘*’ signifies significant differences for comparisons to the control groups, which are empty vector or aerobic culturing with NO_3_^−^ (*P*-value<0.05, *t*-test). At least three biological replicates were performed for each experimental condition. Each data point was denoted by mean value±s.e.

Given the importance of aerobic respiration and reducing power to persistence in stationary phase, we considered the possibility that reactive oxygen species (ROS) may be involved in the process^30^. ROS are by-products of aerobic respiration generated from the inadvertent transfer of electrons to O_2_ instead of intended electron carriers. ROS have the capacity to damage biomolecules including proteins, ribosomes and DNA^35^,^36^,^37^, and reduce cellular culturability^38^. To elucidate whether ROS participate in type I persister formation during stationary phase, catalases and superoxide dismutases (SODs) were overexpressed starting at early stationary phase (*t*=6 h) and persister levels were assessed at 24 h. Interestingly, overexpression of these antioxidant enzymes failed to alter persister levels (Fig. 4a). To confirm that overexpression of *katE* and *katG* produced catalytically functional enzymes, we compared H_2_O_2_ detoxification in strains with overexpressed *katE* and *katG* to uninduced and empty-vector controls. As expected, overexpression of *katE* or *katG* significantly increased the H_2_O_2_ detoxification rates of stationary phase *E. coli* (Supplementary Figs 17). To confirm the catalytic competency of overexpressed *sodA* and *sodB*, we complemented the aerobic growth defect of an SOD-deficient mutant (Δ*sodA*Δ*sodB*Δ*sodC*). A previous study has shown that an SOD-deficient mutant cannot grow as efficiently as WT cells in LB within an aerobic environment^39^, and we show that the *sodA*- and *sodB*-expression systems improve growth of the mutant in an O_2_-dependent manner (Supplementary Fig. 18).

To further establish that the importance of respiration to persister formation is independent of ROS, we performed experiments where cultures were provided with an alternative terminal electron acceptor (nitrate: NO_3_^−^) and transferred to anaerobic conditions at early stationary phase. NO_3_^−^ is used for anaerobic respiration and we postulated that if NO_3_^−^ enhanced persister formation within anaerobic environments it would provide further support that ROS were not involved in the phenomenon and that respiratory activity was the critical process. When NO_3_^−^ was added to cell cultures prior to transferring them to anaerobic conditions (*t*=6 h), we found that anaerobic respiration enhanced type I persister formation (Fig. 4b). Further, when nitrate reductases were inhibited with KCN^40^,^41^, persister formation was reduced to that observed for transfer to anaerobic conditions in the absence of exogenous electron acceptors (Fig. 4b). Overall, these results provide convincing evidence that the importance of respiratory activity to persister formation in stationary phase is not dependent on ROS.

### Respiratory inhibition prevents bacterial self-digestion

**Figure 5:**
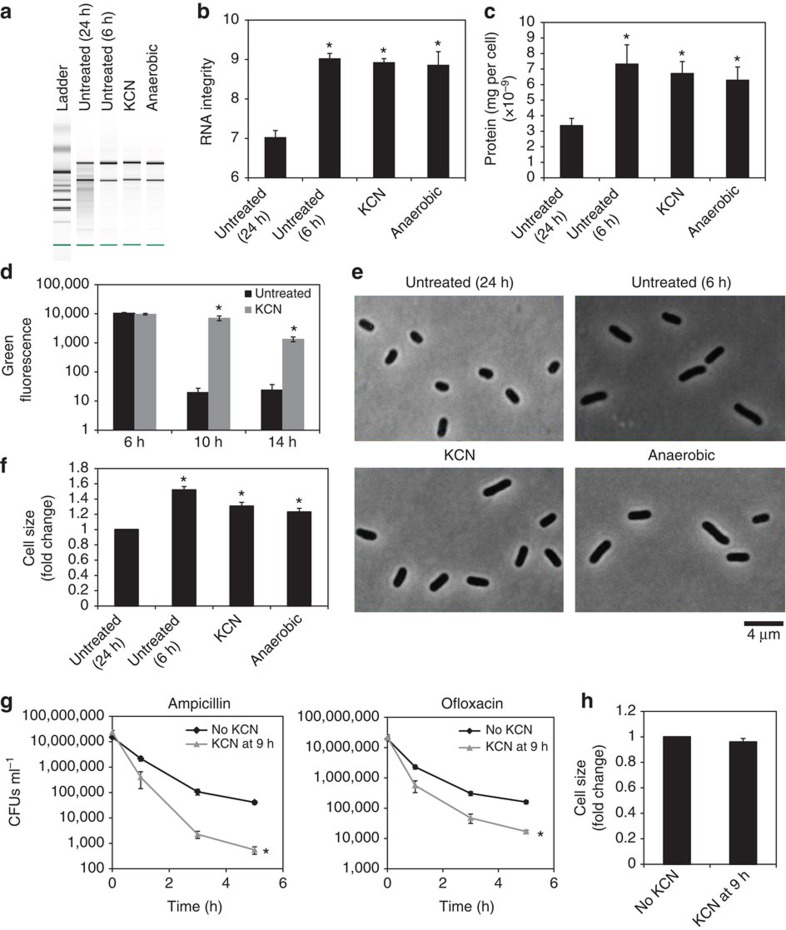
RNA integrity, protein levels and degradation, and cells size of stationary phase cells. (**a**–**c**,**e**,**f**) Cell cultures at early stationary phase (*t*=6 h) were treated with 1 mM KCN or transferred to an anaerobic chamber. At *t*=24 h, cells were pelleted for RNA, protein, and microscope analyses. For controls, untreated overnight cultures (*t*=24 h) and early stationary phase cultures (*t*=6 h) were used. (**a**,**b**) RNA quality was determined with a bioanalyzer using an RNA 6000 Nano kit. The degradation of rRNA was assessed with RNA integrity values which range from 10 (intact) to 1 (totally degraded). (**c**) Cells were sonicated and the protein content in the supernatant was determined with Bradford assays. (**d**) Before KCN treatment at *t*=6 h, the inducer for *gfp* expression was removed in the cultures with the cells carrying pQE-80L*gfp*–*ssrA*. After the KCN treatment, GFP levels were measured. Background fluorescence was determined using cells with empty vectors. (**e,f**) Phase-contrast images of fixed cells were taken using a microscope, and cell size (fold change relative to 24 h untreated overnight cultures) were determined with ImageJ. (**g,h**) KCN treatment was performed at *t*=9 h. At *t*=24 h, microscope images were taken, and ampicillin and ofloxacin persister levels were determined. ‘*’ signifies significant differences for comparisons to control groups (*P*-value<0.05, *t*-test). At least three biological replicates were performed for each experimental condition. Each data point was denoted by mean value±s.e.

The evidence we obtained from KCN-treated and anaerobically transferred cultures, as well as the TCA cycle mutants suggested that respiration, which is powered by reducing equivalents (for example, NADH, UQH_2_), was critical to persister formation during stationary phase. Interestingly, during stationary phase, reducing power is primarily derived from acetate or digestion of endogenous cellular components such as phospholipids, ribosomes, and proteins^42^,^43^. When the persister levels of Δ*ackA*Δ*acs*, which cannot consume acetate^44^,^45^, and Δ*ackA*Δ*poxB*, which cannot produce acetate^46^, were measured, no significant difference was observed between the mutants and WT (Supplementary Fig. 19). This data suggested that the effects of KCN, transfer to anaerobic conditions, and the various TCA cycle mutants on persistence may be mediated by inhibition of self-digestion. To test this hypothesis, we measured persisters in a Δ*fadD*Δ*fadK* mutant, which cannot degrade phospholipids^47^, and measured cell size, protein levels, and rRNA integrity in KCN-treated and anaerobic-transferred stationary phase cells. We note that phospholipid degradation, unlike degradation of ribosomes and proteins, can be readily removed by genetic mutation^47^. As controls, we used untreated cultures and cultures before chemical treatment or transfer to the anaerobic chamber. For Δ*fadD*Δ*fadK*, we did not observe a significant difference in persister levels between the mutant and WT (Supplementary Fig. 19), which suggested that degradation of phospholipids was not essential to persister formation. However, analysis of the electrophoretic traces (Fig. 5a) and RNA integrity values (Fig. 5b) revealed that untreated stationary phase cells have more significantly degraded rRNA compared with KCN-treated, anaerobically transferred or early stationary phase cultures, which yield significantly reduced type I persister levels. Further, cell size (Fig. 5e,f) and overall protein levels (Fig. 5c) in the untreated late stationary phase cells were significantly reduced compared with other groups. To directly measure protein degradation, we employed an IPTG-inducible ssrA-tagged *gfp*. Early stationary phase cultures where ssrA-tagged *gfp* expression had been induced were washed to remove inducer and resuspended in filter-sterilized spent media (without inducer) obtained from WT cultures grown under identical conditions. Cultures were then immediately treated with KCN to inhibit respiration. As depicted in Fig. 5d, KCN treatment significantly reduced the GFP degradation rate when compared with the untreated control. We note that washing and re-suspending the cells in spent media did not alter the effect of KCN on type I persister formation (Supplementary Fig. 20).

As noted above, cells from KCN-treated and anaerobic-transferred cultures were larger than cells from the control (Fig. 5e,f). Reductive division is the major route by which bacteria reduce their size within stationary phase^38^, and therefore, we sought to assess its importance to type I persister formation. To do this, we allowed cells to proceed through reductive division and then determined if the effect of KCN was still present. Although treating cultures with KCN at a later time point (*t*=9 h) resulted in a cell size equivalent to that of untreated cells (Fig. 5h and Supplementary Fig. 21), a significant reduction in persister levels was still observed (Fig. 5g). Collectively, these data demonstrate a clear correlation between respiration, self-digestion, and the formation of type I persisters in stationary phase *E. coli* cultures.

## Discussion

Knowledge of persister physiology enables the identification of elimination strategies^7^,^8^,^48^. For instance, the fact that translation occurs in persisters^22^, though at low levels, led to the discovery that specific metabolites can stimulate aminoglycoside killing of persisters^7^,^11^,^49^. Unfortunately, knowledge of persister physiology remains scarce due to the difficulties associated with segregation of these rare and transient cells from other cell types^11^. Therefore, FACS in conjunction with antibiotic-tolerance assays has become a highly useful method to quantify persister physiology that does not require their segregation from other cell types^11^,^13^,^17^,^24^,^50^,^51^. In these methods a phenotypic quality is fluorescently labelled, the entire bacterial population is sorted into quantiles based on individual cell fluorescence, and the quantiles are treated with antibiotics to enumerate persisters. Using this approach and a fluorescent measure of metabolic activity (RSG) we previously found that persisters from growing populations were highly enriched in the least metabolically active subpopulation due to the lower metabolic activity of the non-growing cells within those cultures^17^. Inspired by this, we applied a similar methodology to study the association between metabolic activity and persisters arising from stationary phase, known as as type I persisters^2^. We discovered that type I persisters largely arose from cells with the highest level of redox activity in this nutrient-depleted environment and were largely absent from the subpopulation with the lowest level. Cells with low redox activity exhibited hallmarks of healthy physiology that included low levels of non-growing and unculturable cells, and rapid resumption of protein synthesis and cell division on exposure to fresh nutrients (Fig. 1). Alternatively, higher levels of stationary phase redox activity produced a higher proportion of cells that were less fit to rapidly resume growth and translation when presented with fresh nutrients. Interestingly, these poorly transitioning cells, which are the source of the majority of persisters in growing populations, have relatively low metabolic activity in growth-promoting environments compared with the cells that have resumed replication^17^. Therefore, an inverse correlation can be drawn between high relative redox activity in the preceding stationary phase and the likelihood of achieving high relative redox activity in the subsequent exponential growth phase.

Given the results obtained with RSG, we hypothesized that respiration was important for type I persister formation. When respiratory functions during stationary phase were inhibited by either chemical (KCN), environmental (anaerobic) or genetic means (Δ*mdh*, Δ*sdhC*, Δ*sucB* and Δ*ubiF*), reduced persister formation was observed and the same hallmarks of healthy physiology observed for the low redox activity subpopulation of RSG-stained cultures were found. These data suggested that respiration could provide a broadly druggable system for the inhibition of persister formation in non-growing, nutrient-starved populations.

As part of our mechanistic investigation into how respiratory inhibition impaired type I persister formation, we considered whether perturbations to ppGpp, Lon or DksA, which have all been previously found to reduce persister levels^16^,^24^,^25^,^52^, produced bacterial populations with similar properties as respiratory-inhibited cultures. ppGpp is a global regulator of bacterial physiology affecting processes such as transcription, DNA replication and cell-wall biogenesis^53^,^54^,^55^. DksA is a transcriptional regulator that works in conjunction with ppGpp to execute the stringent response^56^, whereas ppGpp also stimulates polyphosphate accumulation to activate the Lon protease^16^. Many antitoxins are degraded by Lon, which in turn liberates the associated toxins to produce dormant cells^25^. Given this model, Δ*relA*Δ*spoT* and Δ*lon* reduce persistence through the prevention of toxin-induced stasis^16^,^24^,^25^. Corollaries of these mechanisms include that Δ*dksA*, Δ*relA*Δ*spoT* and Δ*lon* cultures would contain fewer non-growing cells and synthesize protein more quickly than WT. However, we did not find these to be the case; removal of ppGpp, Lon or DksA obviously yields states that are not conducive to the formation of type I persisters, but their abundances of non-growing cells, abilities to translate protein, and oxygen utilization on entry into stationary phase did not significantly differ from those of WT. This is not wholly unexpected, since ppGpp, Lon and DksA are all global regulators and loss of any one of them leads to pleotropic effects; however, it does suggest that further characterization of the states engendered by Δ*relA*Δ*spoT*, Δ*lon* and Δ*dksA* that fail to produce type I persisters is warranted.

On the basis of the collective evidence presented here, we attribute the reduction in type I persister formation of respiratory-inhibited cultures to broad stalling of the processes of self-digestion, which is a mechanism consistent with the prevailing model of persister formation that is based on a specific type of self-digestion (protease-dependent liberation of endoribonuclease toxins)^57^. We note that population-scale measurements are not equivalent to physiological measurements on persisters, but that the data presented (Figs 2, 3 and 5) depict a physiological state of respiratory-inhibited cultures that is poorly suited to achieve the transient tolerance needed to survive exposure to β-lactam and fluoroquinolone antibiotics when presented with fresh nutrients. Though all methods to reach such a state investigated here were associated with respiratory dysfunction, we concur with previous hypotheses that any method to yield such physiology will likely reduce type I persister formation^3^,^19^,^33^,^58^,^59^.

Persistence is a complex phenotype that has been difficult to crack, and many unanswered questions remain regarding the antibiotic tolerances of this highly heterogeneous subpopulation. Despite these uncertainties, processes and mediators important to persistence under different conditions continue to be identified^5^,^7^,^8^,^16^,^18^,^24^,^25^,^30^,^51^,^60^,^61^,^62^,^63^, and methods to translate that knowledge into eradication strategies developed^7^,^8^. Such discoveries provide hope that anti-persister therapies will become a reality in the not too distant future, and with the work presented here, respiration of non-growing bacterial communities now offers another system to target for achievement of such therapeutic goals. Interestingly, others have hypothesized that self-digestion, which is otherwise known as autophagy in eukaryotes, plays an important role in persistence of cancer cell populations by inducing cell dormancy under unfavourable conditions^64^,^65^. It is tempting to draw parallels between persistence of bacteria and cancer cells to their respective therapeutics^66^,^67^, and muse that knowledge of each will enrich investigations of the other.

## Methods

### Bacterial strains

Strains used in this study were generated from *E. coli* MG1655 (ATCC 700926)^68^. MO001, which has a chromosomally integrated *lacI*^*q*^ promoter in place of the *lacI* promoter and a chromosomally integrated *T5p*-*mCherry* in place of *lacZYA*, was generated in a previous study^17^. A pQE-80L plasmid variant (Qiagen, Valencia, CA), which has a synthetic *T5* promoter, *lacI* expressed from the *lacI*^*q*^ promoter, and confers kanamycin resistance, was used for gene overexpression. All primers used to generate pQE-80L*mCherry*, pQE-80L*gfp*, pQE-80L*gfp*–*ssrA*, pQE-80L*katE*, pQE-80L*katG*, pQE-80L*sodA*, and pQE-80L*sodB*, as well as descriptions of the cloning steps, are presented in Supplementary Table 1. Genetic deletions (Δ*sucB*, Δ*sdhC*, Δ*mdh*, Δ*icdA*, Δ*acnB*, Δ*phoU*, Δ*ubiF*, Δ*lon*, Δ*dksA*, Δ*tolC*, Δ*relA*Δ*spoT*, Δ*fadD*Δ*fadK*, Δ*ack*Δ*acs*, Δ*ack*Δ*poxB*, and Δ*sodA*Δ*sodB*) were transduced from strains within the Keio collection^69^, or strains that were constructed previously^24^,^52^ using the standard P1 phage method^69^. When necessary the kanamycin-resistance gene (*Kan*^*R*^) was removed using FLP recombinase^70^. Due to the proximity of *sodC* to *sodB*, P1 phage method was not used to delete *sodC*. Instead, *sodC* was deleted from the chromosome of Δ*sodA*Δ*sodB* strain using the method of Datsenko and Wanner^70^. All mutations and plasmid insertions were confirmed using PCR and/or DNA sequencing (Genewiz, South Plainfield, NJ).

### Chemicals, media, and growth conditions

Unless noted below, all chemicals were purchased from Fisher Scientific or Sigma Aldrich. RSG and PI were purchased from Life Technologies, Invitrogen (Grand Island, NY), IPTG was purchased from Gold Biotechnology (St Louis, MO), and fluorescent particles for cell counts were purchased from Spherotech, Inc. (Lake Forest, IL). LB medium (10 g l^−1^ Tryptone, 5 g l^−1^ Yeast Extract, and 10 g l^−1^ NaCl) and LB agar plates (LB+15 g l^−1^ agar) were prepared from components, and used for planktonic growth and enumeration of c.f.u., respectively. To prepare M9 salt solution, 2 ml of 1 M MgSO_4_ and 100 μl and 1 M CaCl_2_ were first mixed with 800 ml distilled water, then 200 ml of 5 × M9 salt solution (33.9 g l^−1^ dibasic sodium phosphate, 15 g l^−1^ monobasic potassium phosphate, 5 g l^−1^ ammonium chloride, and 2.5 g l^−1^ sodium chloride). For persister assays, final concentrations of 200 μg ml^−1^ ampicillin^15^ and 5 μg ml^−1^ ofloxacin^20^ were used. For selection, 50 μg ml^−1^ kanamycin and 100 μg ml^−1^ ampicillin were used. To induce protein expression, 1 mM IPTG was used. M9 media and all chemical solutions were filter-sterilized with 0.22-μm filters. LB and LB agar were sterilized by autoclaving. Overnight cultures were prepared from a 25% glycerol, −80 °C stock in 2 ml LB medium in a test tube and cultured at 37 °C with shaking (250 r.p.m.) for 24 h. After specified treatments (see below), stationary phase cells were diluted 100-fold in 1 ml of fresh LB in a test tube for persister, cell division and protein expression assays.

### Staining with redox sensor green and propidium iodide

Overnight cultures were filter sterilized using 0.22-μm filters to obtain cell-free spent medium. Then, stationary phase cells were diluted in the spent media to obtain ∼10^7^ cells per ml, which was necessary to prevent potential clogging of the sorter. Then, diluted samples were stained with RSG at 1 μM concentration and incubated at 37 °C with shaking for ∼1 h. As a control, cells were diluted in spent media, and cultured for 1 h at the same conditions described above without staining. To ensure that RSG fails to stain metabolically inactivated cells, ∼10^7^ cells per ml stationary phase cells were killed in 70% ethanol solution for ∼1 h, and then cells were centrifuged for 3 min at 15,000 r.p.m. (21,130*g*, Eppendorf 5424), and the supernatant was removed. Then the cell pellet was resuspended in filter-sterilized spent media and RSG staining was performed as described above. To enhance stationary phase metabolic activity, stationary phase cells were treated with 20 mM glucose for 1 h before RSG staining. Then, metabolically stimulated cells were stained with RSG as described above. We note that, since RSG fluorescence does accumulate as a function of time, all samples were strictly stained for the same amount of time and interpretation of RSG data was always based on relative fluorescence.

For PI staining, diluted samples were stained with PI at 30 μM concentration and incubated at 37 °C in the dark for 15 min. As a positive control, stationary phase cells were killed in 70% ethanol solution for ∼1 h, and then cells were centrifuged for 3 min at 21,130*g*, the supernatant was removed, and cell pellet resuspended in PBS. These dead cells were then stained with PI as described above. All stained and unstained samples were used for FACS analysis.

### Inhibition of stationary phase metabolism and macromolecular synthesis

To inhibit cytochrome oxidase and nitrate reductase activities, KCN (0.5, 1.0 and 2.0 mM) was used. For inhibition of aerobic respiration, cells were transferred to an anaerobic environment (Coy Hypoxic Glovebox with Anoxic Upgrade, Grass Lake, MI). When necessary, 40 mM NaNO_3_ was used in the cell cultures before transferring cells into an anaerobic chamber. RIF and CAM were used at concentrations of 25, 50 and 100 μg ml^−1^ to inhibit RNA and protein synthesis, respectively. Overnight cultures were prepared as described above, and treated with chemical inhibitors or transferred to the anaerobic chamber at early (*t*=6 h) or late stationary phase (*t*=22 h). As controls, cultures were treated with solvents used to dissolve the inhibitors: H_2_O for KCN-treated samples or DMSO for CAM- and RIF-treated samples. At *t*=24 h, cells were washed to remove inhibitors. To do this, cultures were centrifuged for 3 min at 21,130*g*, the supernatant was removed, and cell pellet resuspended in 1 ml of fresh LB. Washed cells were diluted 100-fold in 1 ml of fresh media for persister, protein expression and cell division assays.

### Catalase and superoxide dismutase overexpression

Overnight cultures of *E. coli* strains carrying pQE-80L with specified genes were treated with 1 mM IPTG to induce protein production at *t*=6 h. At *t*=24 h, cells were washed to remove the inducer and diluted 100-fold in fresh media for persister assays. For controls, *E. coli* cells carrying an empty vector (pQE-80L without a gene) were used.

To demonstrate that overexpression of *katE* and *katG* produced catalytically functional enzymes, H_2_O_2_ detoxification in strains with overexpressed *katE* and *katG* were compared with uninduced and empty-vector controls. Overnight cultures of *E. coli* strains carrying empty vector or pQE-80L with specified *katE* and *katG* were treated with 1 mM IPTG to induce protein production at *t*=6 h. At *t*=8 h, cells were washed to remove the inducer, and then diluted in M9 salt media to OD_600_∼0.1 in test tubes. Cell cultures were then treated with 2 mM H_2_O_2_, and incubated at 37 °C with shaking. H_2_O_2_ concentrations at indicated time points were measured using an Amplex Red Assay (Life Technologies) as described previously^44^.

To demonstrate the catalytic competency of overexpressed *sodA* and *sodB*, the plasmids carrying these genes were transferred to SOD-deficient mutant (Δ*sodA*Δ*sodB*Δ*sodC*). Overnight cultures of WT and SOD-deficient mutants carrying the plasmids were incubated in 2 ml LB medium with 40 mM NaNO_3_ in a test tube and cultured at 37 °C with shaking (250 r.p.m.) for 24 h in an anaerobic chamber. Then, the overnight cultures were diluted to OD_600_∼0.05 in 10 ml LB with 1 mM IPTG to induce *sodA* and *sodB* expression in 250-ml buffled flasks to increase the aeration, and cultured aerobically at 37 °C with shaking (250 r.p.m.). For the controls, the overnight cultures were similarly diluted to OD_600_∼0.05 in 10 ml LB with 1 mM IPTG+40 mM NaNO_3_ in 250-ml buffled flasks, and cultured anaerobically at 37 °C with shaking (250 r.p.m.). At indicated time points, growth was measured by OD_600_ with a Synergy H1 Hybrid Multi-Mode microplate reader (BioTek, Winooski, VT).

### Dissolved oxygen measurements

Dissolved oxygen concentrations were measured using the FireStingO_2_ fibre-optic O_2_ meter with the OXYROB 10-CL2 robust oxygen miniprobe (PyroScience, GmbH). To enable comparable culturing to other assays used (2 ml of media at 37 °C with shaking), oxygen concentrations could not be monitored continuously, but rather had to be quantified at specified time points. To do this, samples were briefly removed from the shaking incubator and the oxygen probe was inserted into the media. Stable oxygen concentrations were obtained within 2 min of insertion of the probe into LB medium without cells and WT cultures at 6 h, which rapidly consumed oxygen until only trace levels remained (Supplementary Fig. 22). Therefore, oxygen levels in all cultures were reported exactly 2 min after insertion of the probe into cultures (Supplementary Fig. 22). We note that these measurements reflect relative respiratory activity, rather than exact oxygen concentrations under the culturing conditions used, because removal of cultures from shaking at the cell densities used reduces the oxygen transport into the media.

### Cell sorting

Cells were sorted using a FACSVantage SE w/DiVa (BD Biosciences, San Jose, CA) cell sorter at 16 p.s.i. with a 70-micron nozzle. Cell populations were determined using forward and side scatter parameters (FSC and SSC), and green fluorescence (RSG) was identified using 488-nm excitation with 530/30 bandpass filter. Sterile PBS buffer was used as sheath fluid in the sorter. After determination of the regions to be sorted, ∼200,000 cells from each subpopulation were collected, and each sorting procedure lasted 15 min or less. Since the cells were collected in sheath fluid (PBS), the sorted samples were immediately mixed 1:1 with a rich medium (2 × Tryptone, 2 × Yeast Extract, and 1 × NaCl), which yielded a medium similar to LB. This procedure generated ∼1-ml samples. Samples were then used for persister, cell division and protein expression assays.

### Flow cytometry analysis

Fluorescent protein levels were analysed with an LSRII flow cytometer (BD Biosciences, San Jose, CA). Cell populations were detected using forward and side scatter parameters (FSC and SSC), and fluorescence was assayed with a laser emitting at 488 nm for GFP and 561 nm for mCherry. Fluorescence was collected using green (525/50 nm) and red (610/20 nm) fluorescence bandpass filters. Data were acquired and analysed using FACSDiVa software (BD Biosciences, San Jose, CA) and FlowJo (Tree Star Software, Ashland, OR). Counting beads, which were detected by FSC and SSC parameters, were used to enumerate cells.

### Cell division assay

MO001 cells and its derivatives were cultured as described above with 1 mM IPTG to express *mCherry*. When necessary, overnight cultures were treated with chemical inhibitors or transferred to anaerobic chamber at *t*=6 h. At 24 h, cells were washed to remove IPTG and inhibitors if used. Cell pellets were either suspended in spent media for RSG staining and FACS analysis or diluted (100-fold) in 1 ml of fresh media as described above. Sorted or diluted samples were cultured without inducer for 2.5 h at 37 °C and 250 r.p.m. Cell cultures at *t*=0 h and 2.5 h were washed and suspended in PBS and analysed immediately with a flow cytometer to quantify mCherry levels. The gates of the non-growing population, whose mCherry levels remained high, were determined using the overnight cell cultures (Supplementary Fig. 23). Non-growing cells and the entire population were enumerated with counting beads.

### Fluorescent protein expression assay

Stationary phase cells carrying pQE-80L plasmids with a fluorescent protein (mCherry or GFP) were used. Sorted or non-sorted samples were diluted in 1 ml of fresh media with 1 mM IPTG to induce fluorescent protein production. Cells were cultured at 37 °C and 250 r.p.m. for only a short period of time (10 min) to eliminate any effect from cell proliferation. At *t*=0 and 10 min, cells were pelleted to remove the supernatant and suspended in PBS for flow cytometer analysis.

### Persister assay

Cell cultures (1 ml) were treated with either ampicillin (200 μg ml^−1^) or ofloxacin (5 μg ml^−1^) and incubated in a shaker at 37 °C and 250 r.p.m. for 5 h. Samples at designated time points were washed with PBS twice, followed by resuspension in 100 μl of PBS. Ten microlitres of the sample was serially diluted in PBS and spotted on LB agar to measure c.f.u. To increase the limit of detection, the remaining 90 μl sample was also plated on LB agar. The frequency of persisters was calculated as the ratio of the number of persisters in a sample to the initial number of total cells before antibiotic treatment. Recovery was calculated from the persister frequencies of the quantiles segregated with FACS as described in a previous study^17^. This was used to demonstrate the effects of segregation on persister levels since recovery should equal the frequency of persisters obtained from a non-segregated sample.

### RNA isolation

Approximately 250 μl of indicated cultures were mixed with RNAprotect to stabilize RNA. Total RNA was purified with RNeasy extraction kit using the manufacturer’s protocol (Qiagen). On-column DNA digestion with DNase I (Qiagen) was used to remove DNA from samples. Quality of total RNA was assessed with a bioanalyzer using an RNA 6000 Nano kit (Agilent Technologies, Inc, Santa Clara, CA).

### Bradford assay

Bradford protein assays were used to measure protein content. Cultures (1 ml) were washed and suspended in PBS, and then sonicated at 10% amplitude for 20 min. This method lysed ∼99.9% of cell population, which was confirmed by measuring the c.f.u counts before and after the sonication. This procedure did not alter results of the Bradford assay, which was confirmed by measuring the protein concentration of a standard bovine serum albumin (BSA) solution before and after sonication. Sonicated samples were centrifuged 3 min at 21,130*g*, to discard insoluble fraction. Then, 5 μl of supernatant was mixed with 250 μl Bradford buffer in a 96-well flat bottom plate. After incubation for 15 min at room temperature and in the dark, colorimetric change was detected by measuring the absorbance at 595 nm using a plate reader. Standard curves were prepared using standard BSA solutions (1, 0.5, 0.25, 0.125 and 0 mg ml^−1^). Protein concentration was reported as mg per cell, and exact cell counts before the sonication were enumerated with flow cytometry and counting beads.

### Measuring degradation of ssrA-tagged GFP

Cells carrying pQE-80L*gfp*–*ssrA* were cultured in 2 ml LB as described above with 1 mM IPTG to express GFP. At *t*=6 h, cells were washed to remove the inducer and suspended in 2-ml filter-sterilized spent media (without inducer) from WT cultures grown under the same conditions. Then, cells were either treated with 1 mM KCN in sterile H_2_O, or dosed with an equivalent volume of sterile H_2_O without KCN (untreated). At indicated times, GFP levels were measured using a plate reader. Cell cultures containing empty vectors were used to determine the background fluorescence.

### Microscopy imaging

Stationary phase cells were washed and suspended in 4% paraformaldehyde (PFA) in PBS for ∼20 min to fix the cells. Then cells were washed to remove the PFA and diluted fivefold in PBS. Cell suspensions were mixed to prevent formation of cell clumps. Then, cells were stabilized on 1% agarose pads for imaging as described previously^17^. Phase-contrast images were taken with a Nikon Ti-E microscope (Nikon, Melville, NY) equipped with 100 × /1.40 numerical aperture phase objective and NIS Elements software. Cells from the microscope images (without cell-clusters) were analysed to determine the mean value of cell size for each biological replicate using ImageJ (NIH, Maryland, USA), and three independent mean values from three biological replicates for each experimental condition were used for statistical analyses.

### Statistical analysis

At least three biological replicates were performed for each experimental condition. Each data point was denoted by mean value±s.e. A two-tailed *t*-test was performed for pairwise comparisons with unequal variance, and *P*-value≤0.05 was considered significant.

## Additional information

**How to cite this article:** Orman, M. A. & Brynildsen, M. P. Inhibition of stationary phase respiration impairs persister formation in *E. coli*. *Nat. Commun.* 6:7983 doi: 10.1038/ncomms8983 (2015).

## Supplementary Material

Supplementary Information Supplementary Figures 1-23 and Supplementary Table 1
