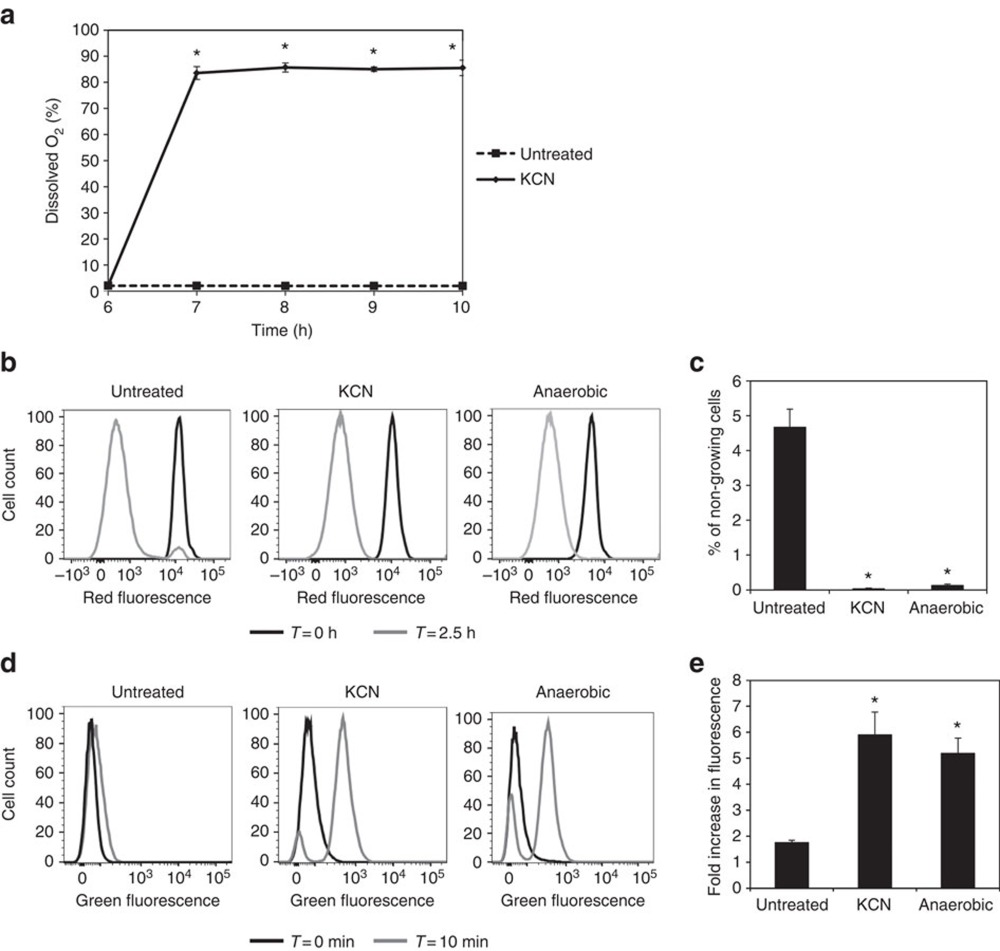# Erratum: Inhibition of stationary phase respiration impairs persister formation in *E. coli*

**DOI:** 10.1038/ncomms10756

**Published:** 2016-02-17

**Authors:** Mehmet A. Orman, Mark P. Brynildsen

Nature Communications **6**: Article number:798310.1038/ncomms8983 (2015); Published: 08062015; Updated: 02172016.

In Fig. 3d in this Article, the graph depicting flow cytometry of the anaerobic culture was inadvertently duplicated from that depicting the KCN-treated culture during the production process. The correct version of Fig. 3 appears below.

**Figure 3**