# Supplementary material for: Inhibition of stationary phase respiration impairs persister formation in E. coli
Source: Nat Commun. 2015 Aug 6;6:7983. doi: 10.1038/ncomms8983 (PMC4530465; doi:10.1038/ncomms8983)
Supplement: Supplementary Information — Supplementary Figures 1-23 and Supplementary Table 1 [file ncomms8983-s1.pdf]

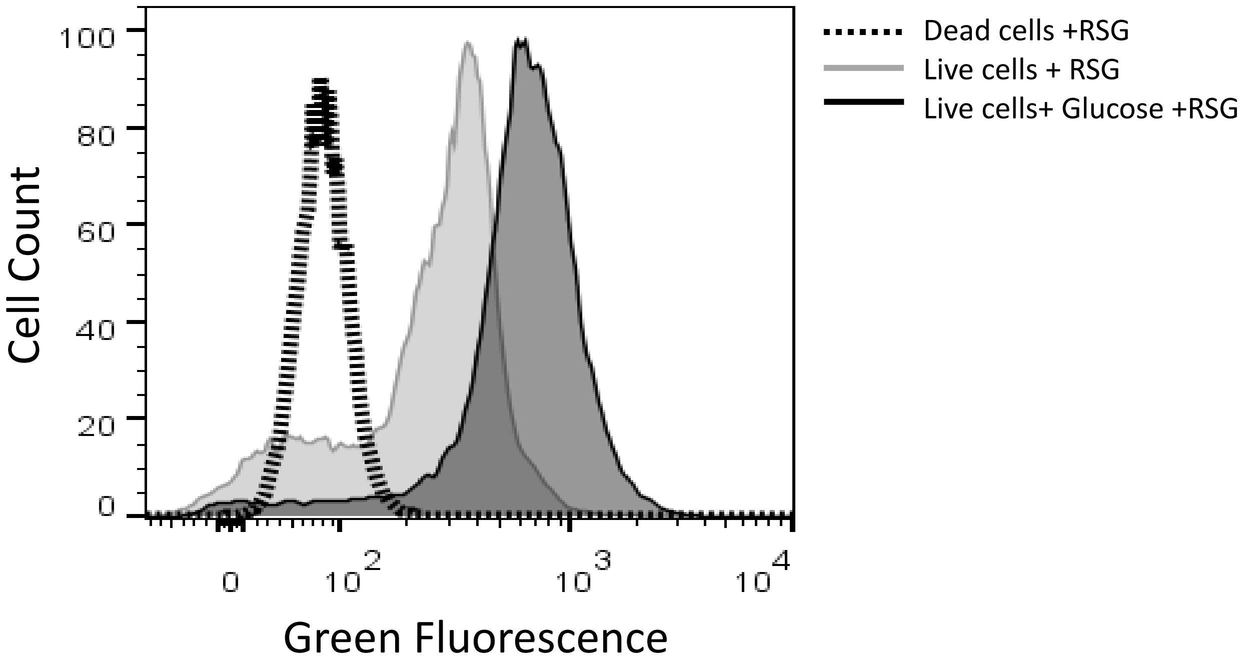

Supplementary Figure 1. RSG staining of live, dead, and metabolically-stimulated cells.

Live, dead, and metabolically-stimulated live cells were identically treated with RSG, and a flow cytometer was used to measure fluorescence. To stimulate stationary phase metabolism, stationary phase cells were treated with 20 mM glucose for 1 h prior to RSG staining. To produce metabolically-inactivated cells, stationary phase cells were treated with 70% ethanol for approximately 1 h to obtain dead cells. At least three biological replicates were performed for each experimental condition.

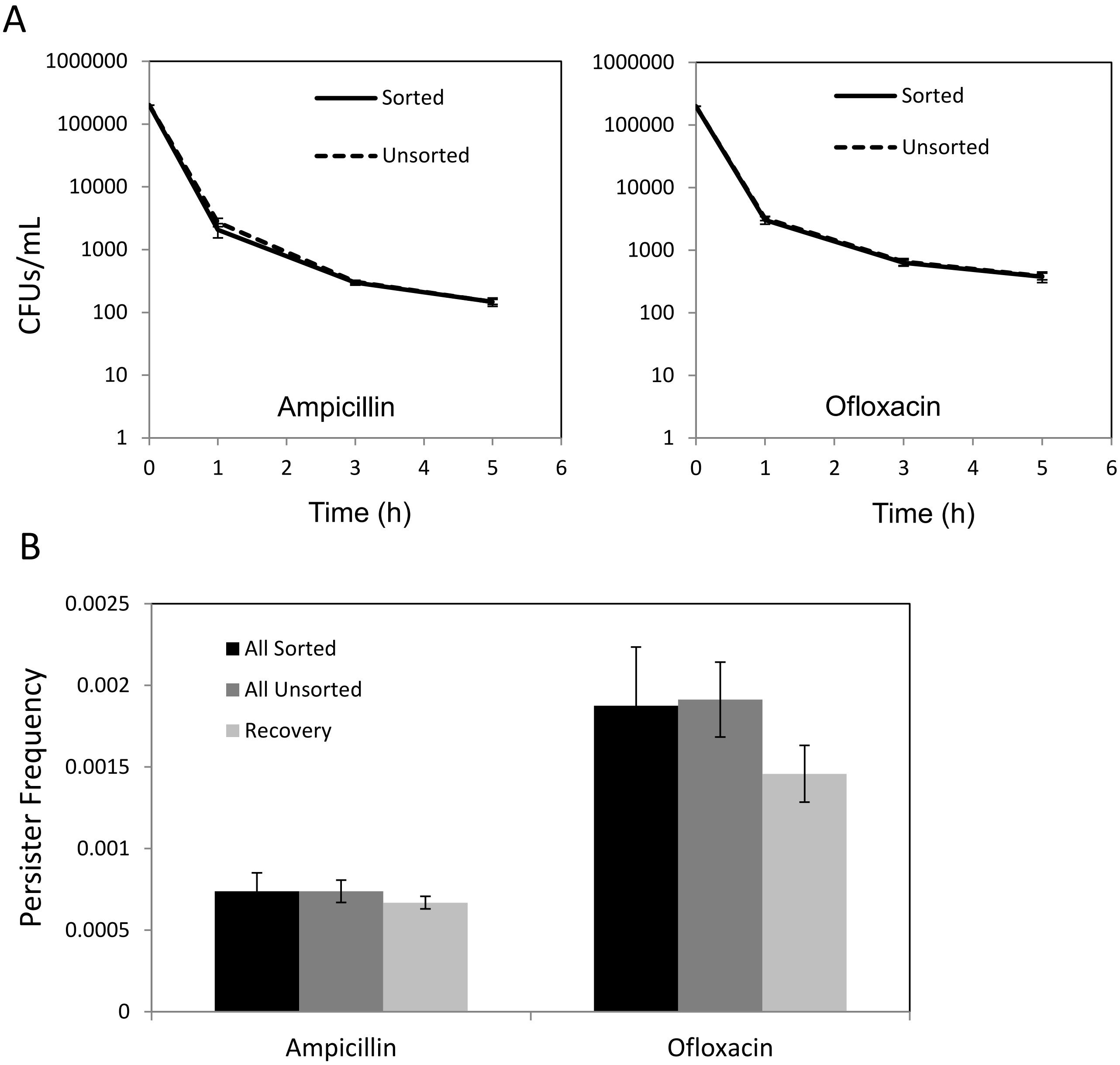

Supplementary Figure 2. Kill curves and FACS controls.

(A) Sorted and unsorted cells from entire population were diluted in fresh LB and treated with ampicillin or ofloxacin for 5h, and CFU levels were monitored by plating at indicated time points. **(B)** Persister frequencies were calculated as the ratio of the number of persisters to the initial number cells before antibiotic treatment. Recovery is a persister frequency for the entire population calculated as a weighted average of the persister frequencies from the quantiles, and it serves as an additional control (Methods). At least three biological replicates were performed for each experimental condition. Each data point was denoted by mean value ± standard error.

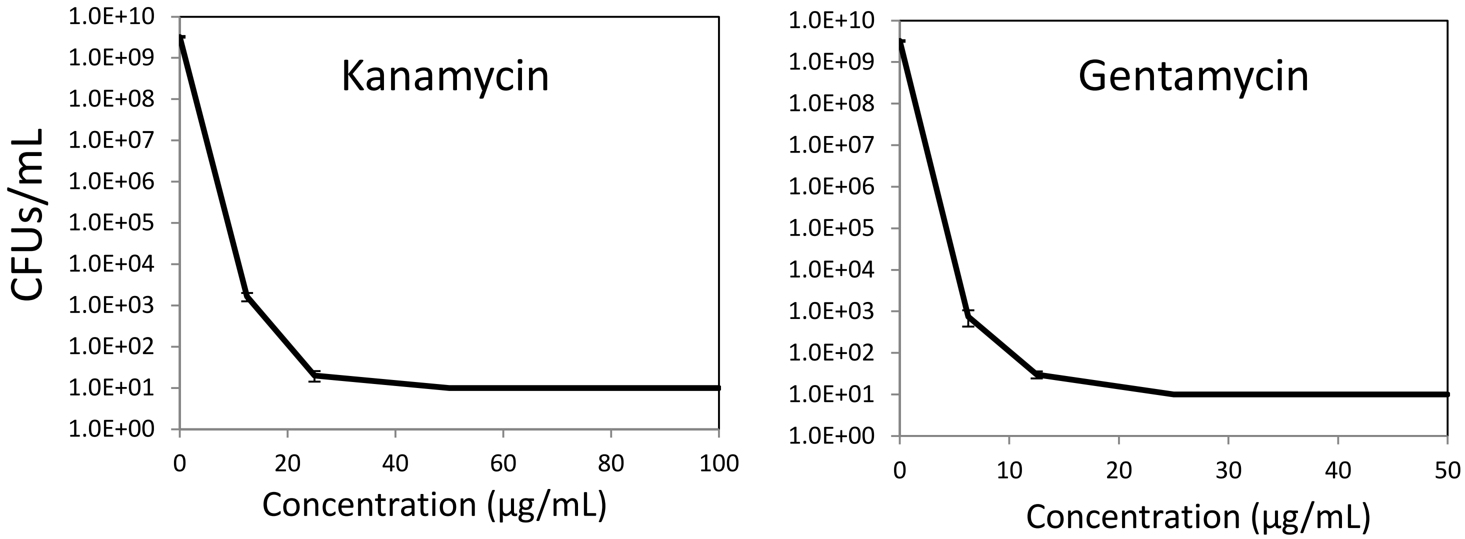

Supplementary Figure 3. Aminoglycoside treatments.

Overnight cultures were diluted in fresh media with kanamycin or gentamicin at indicated concentrations and cultured for 5 h, and CFU levels were monitored by plating after 5 h treatment. The limit of detection of the assay is ~10 CFU/mL. At least three biological replicates were performed for each experimental condition. Each data point was denoted by mean value ± standard error.

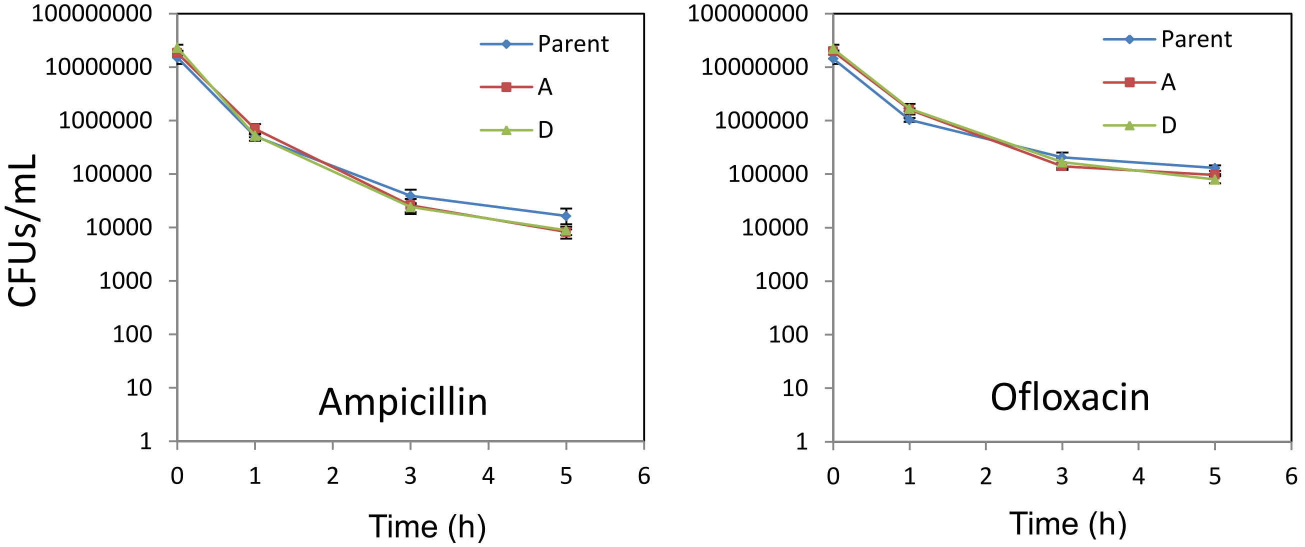

Supplementary Figure 4. Persister levels of stationary phase cultures prepared from parental strain and FACS-sorted A and D subpopulations.

Approximately 200,000 cells from A or D subpopulations were inoculated into fresh media and cultured to stationary phase. Then, these stationary phase cultures were diluted in fresh media and treated with ampicillin or ofloxacin for 5 h, and CFU levels were monitored by plating at the indicated time points. Persister levels in the A- and D-originated cultures were indistinguishable from those of the parental strain. At least three biological replicates were performed for each experimental condition. Each data point was denoted by mean value ± standard error.

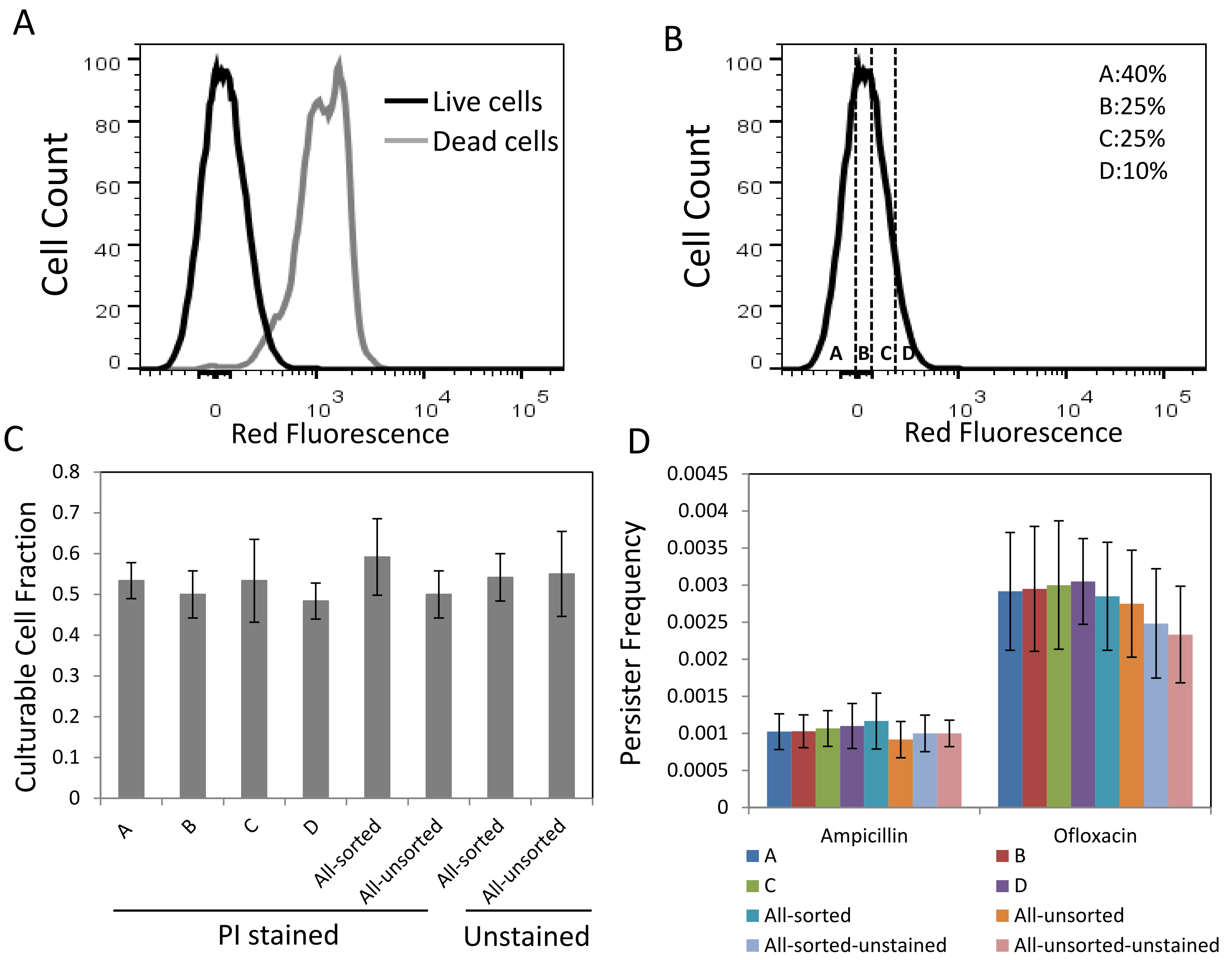

Supplementary Figure 5. PI staining and persistence.

(A) Stationary phase cells with (dead) and without (live) ethanol treatment were stained with PI and analyzed with FACS. Note that PI staining occurs in a threshold manner **(B)** Stationary phase cells were segregated with FACS into four quantiles, A, B, C, and D. We note that the A subpopulation contained approximately 40% of the total population and this was associated with the ability of the sorter to resolve low fluorescing events. **(C)** Shortly after sorting, segregated samples with known abundances of cells were plated on LB agar to enumerate CFUs and calculate culturable cell fractions. **(D)** Segregated samples diluted in fresh LB were treated with ampicillin or ofloxacin for 5 h to determine the persister frequencies. Controls include unsorted and/or unstained samples. Initial cell quantities in unsorted samples were determined with counting beads and a flow cytometer (Methods). At least three biological replicates were performed for each experimental condition. Each data point was denoted by mean value ± standard error.

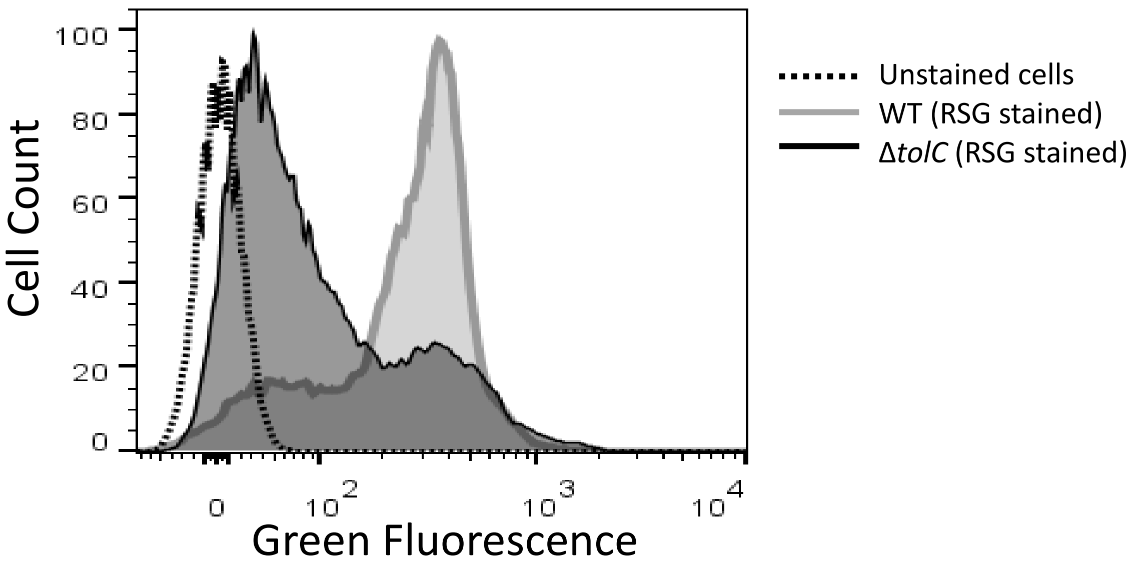

Supplementary Figure 6. RSG staining of Δ*tolC*.

Stationary phase WT and Δ*tolC* cells were stained with RSG and analyzed by flow cytometry. At least three biological replicates were performed for each experimental condition.

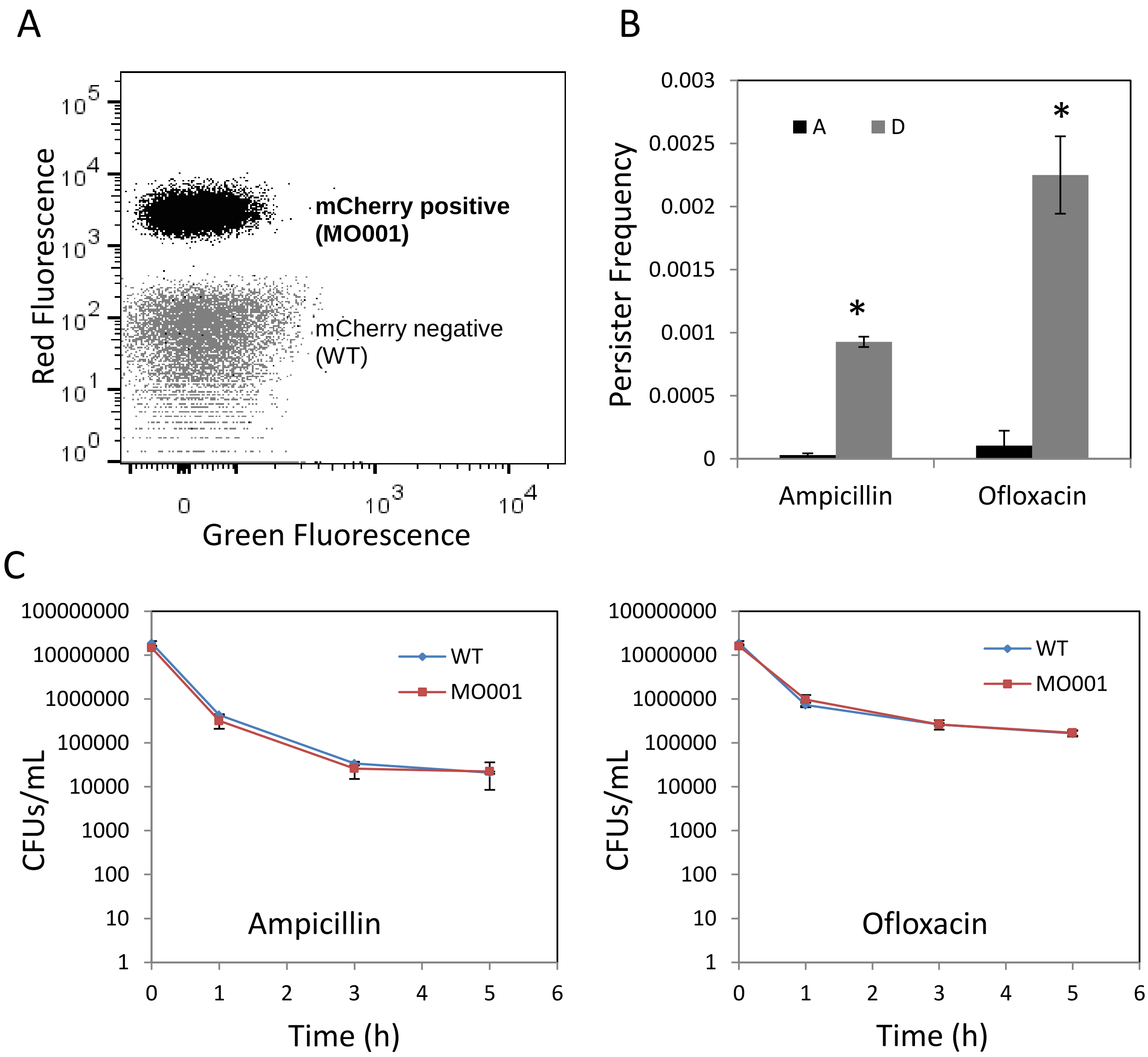

Supplementary Figure 7. FACS and persister analysis of MO001.

WT and MO001 were cultured with 1mM IPTG during overnight growth. **(A)** Red and green fluorescence were measured with a flow cytometer. The MO001 sample comprises the unstained control for Figure 1D. (**B**) After mCherry positive cells from stationary phase cultures (MO001 + IPTG) were stained with RSG, A and D subpopulations were segregated with FACS, and diluted in fresh LB with antibiotics. CFU levels were determined by plating at t=5 h after the treatment. *Note that persister levels in D subpopulations are significantly higher than the persister levels in A subpopulations (p-value<0.05, t-test). (**C**) mCherry positive cells were diluted in fresh media without sorting and treated with antibiotics. CFUs were monitored for 5 h to determine the persister levels. Similarly, persister levels in WT cultures were determined. At least three biological replicates were performed for each experimental condition. Each data point was denoted by mean value ± standard error.

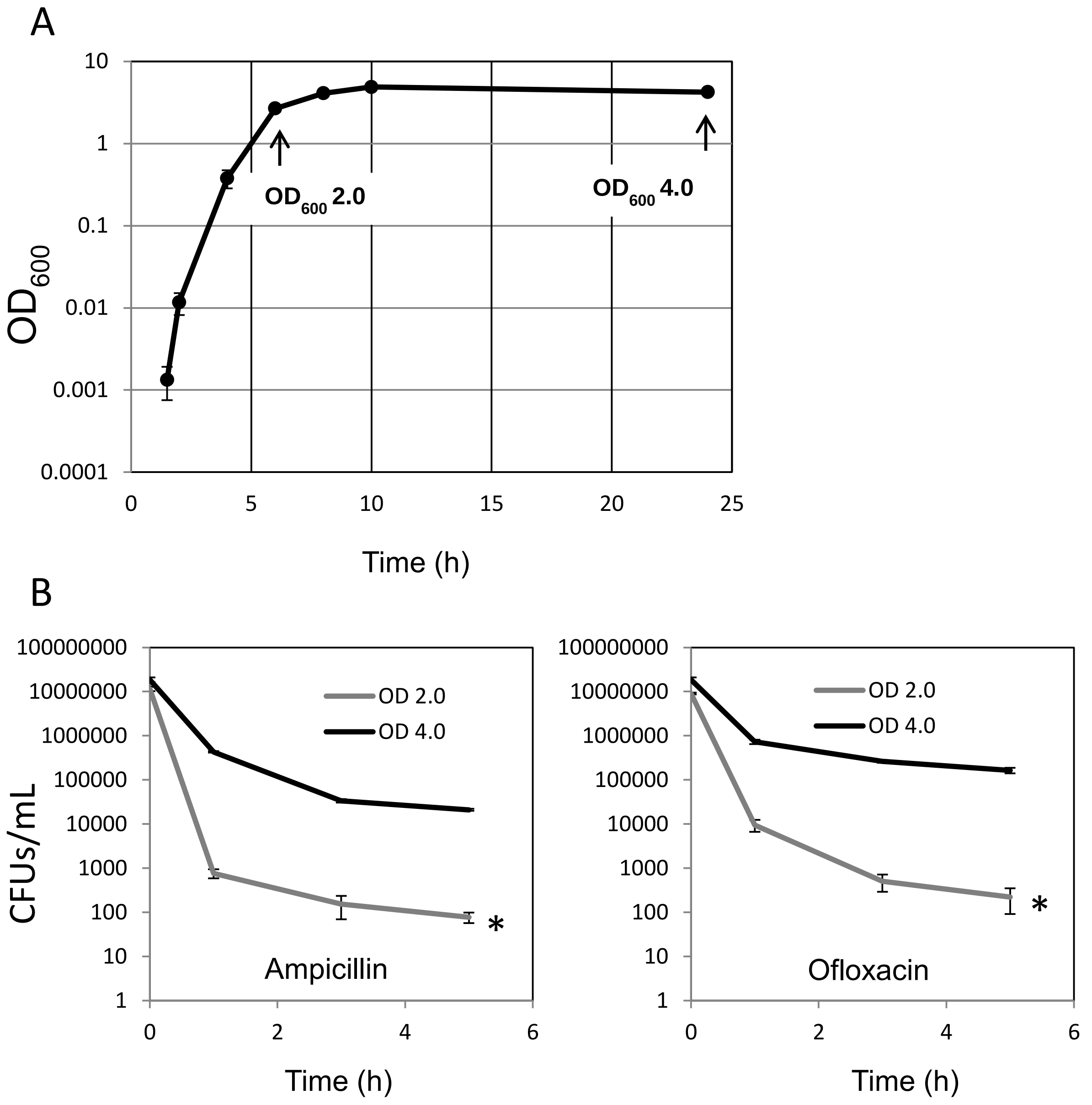

Supplementary Figure 8. Persister levels in early and late stationary phases.

(A) Cells were cultured in 2 mL LB medium in a test tube at 37 ºC with shaking (250 rpm). OD_{600} measurements were taken at indicated time points. **(B)** Cells at early stationary phase (t=6 h) or late stationary phase (t=24 h) were diluted in fresh LB and treated with ampicillin or ofloxacin for 5 h to enumerate persisters. *Note that persister levels in early stationary phase cultures are significantly less than persister levels in late stationary phase cultures (p-value<0.05, t-test). At least three biological replicates were performed for each experimental condition. Each data point was denoted by mean value ± standard error.

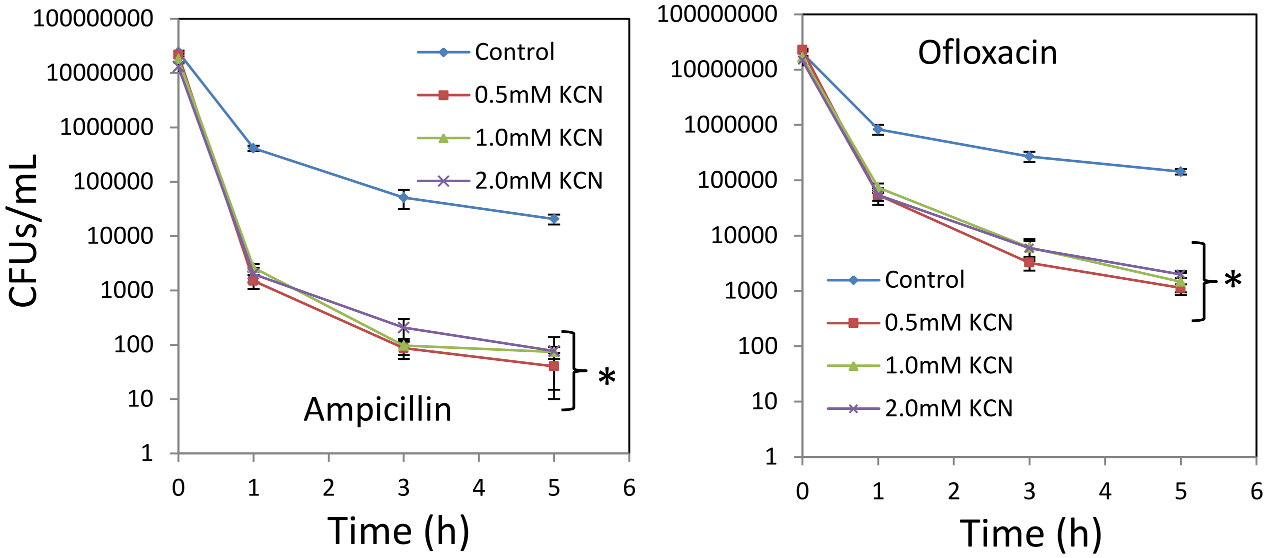

Supplementary Figure 9. Persister levels in cultures treated with KCN at early stationary phase.

Overnight cultures were treated with KCN at indicated concentrations at early stationary phase (t=6 h), and at t=24 h cells were washed, diluted in fresh LB, and treated with ampicillin or ofloxacin for 5 h to monitor the CFU levels. *Note that KCN treatment at all concentrations significantly reduced the persister levels compared to control group (p-value<0.05, t-test). At least three biological replicates were performed for each experimental condition. Each data point was denoted by mean value ± standard error.

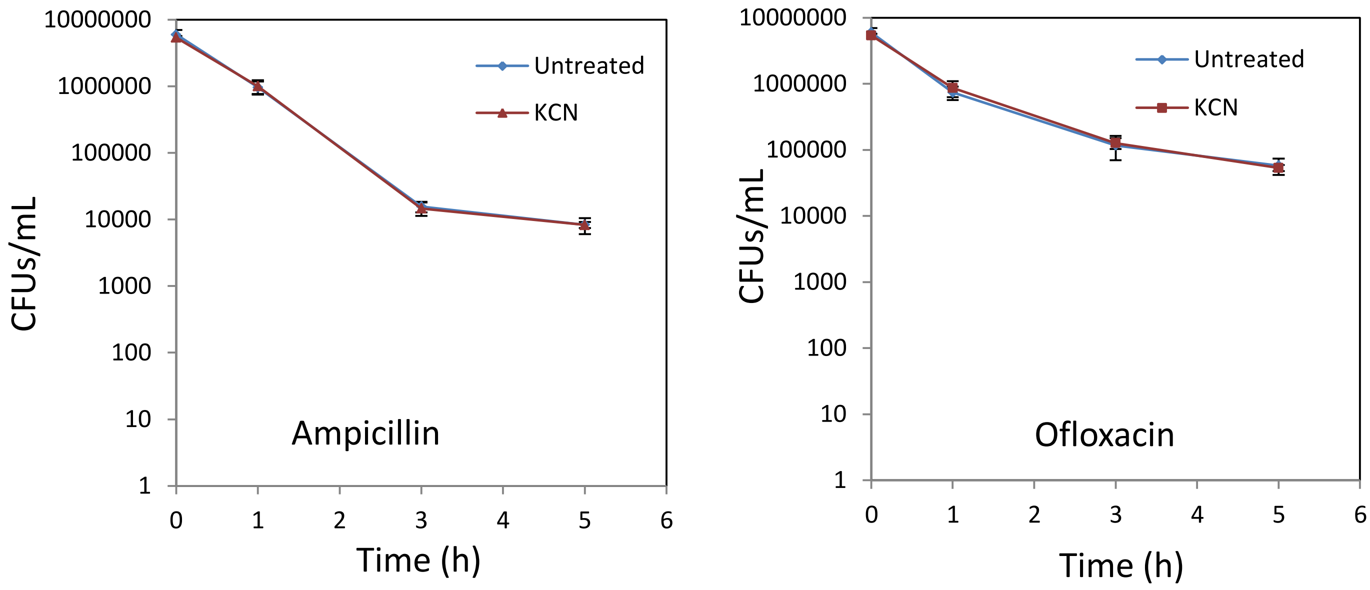

Supplementary Figure 10. Persister levels in cultures treated with KCN at late stationary phase.

Overnight cultures were treated with 1 mM KCN at late stationary phase (t=22 h), and at t=40 h cells were washed, diluted in fresh LB, and treated with ampicillin or ofloxacin for 5 h to monitor the CFU levels. At least three biological replicates were performed for each experimental condition. Each data point was denoted by mean value ± standard error.

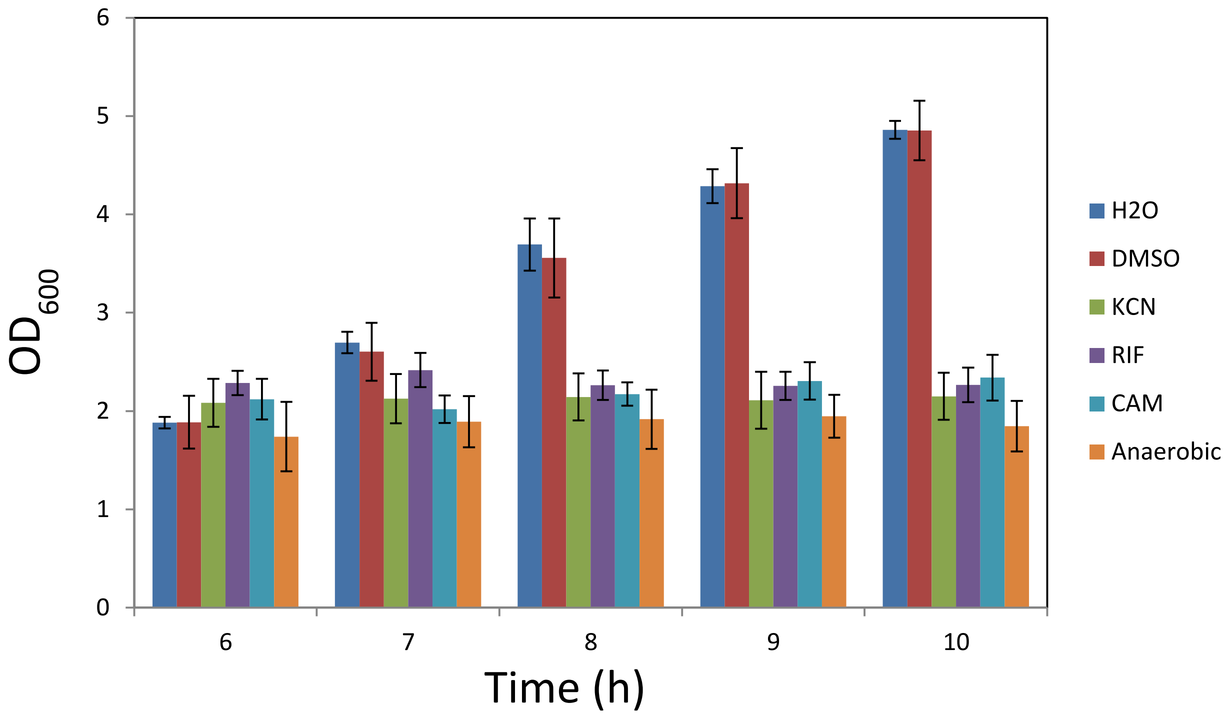

Supplementary Figure 11. Cell growth inhibition by chemical treatments.

Overnight cultures were treated with 1 mM KCN, 50 µg/mL RIF, 50 µg/mL CAM or transferred to an anaerobic chamber at t=6 h, and OD_{600} measurements were performed using a plate reader. Note that chemical treatments or transfer to anaerobic conditions significantly reduced cell growth compared to control groups (H_2O and DMSO) (p-value<0.05, t-test). At least three biological replicates were performed for each experimental condition. Each data point was denoted by mean value ± standard error.

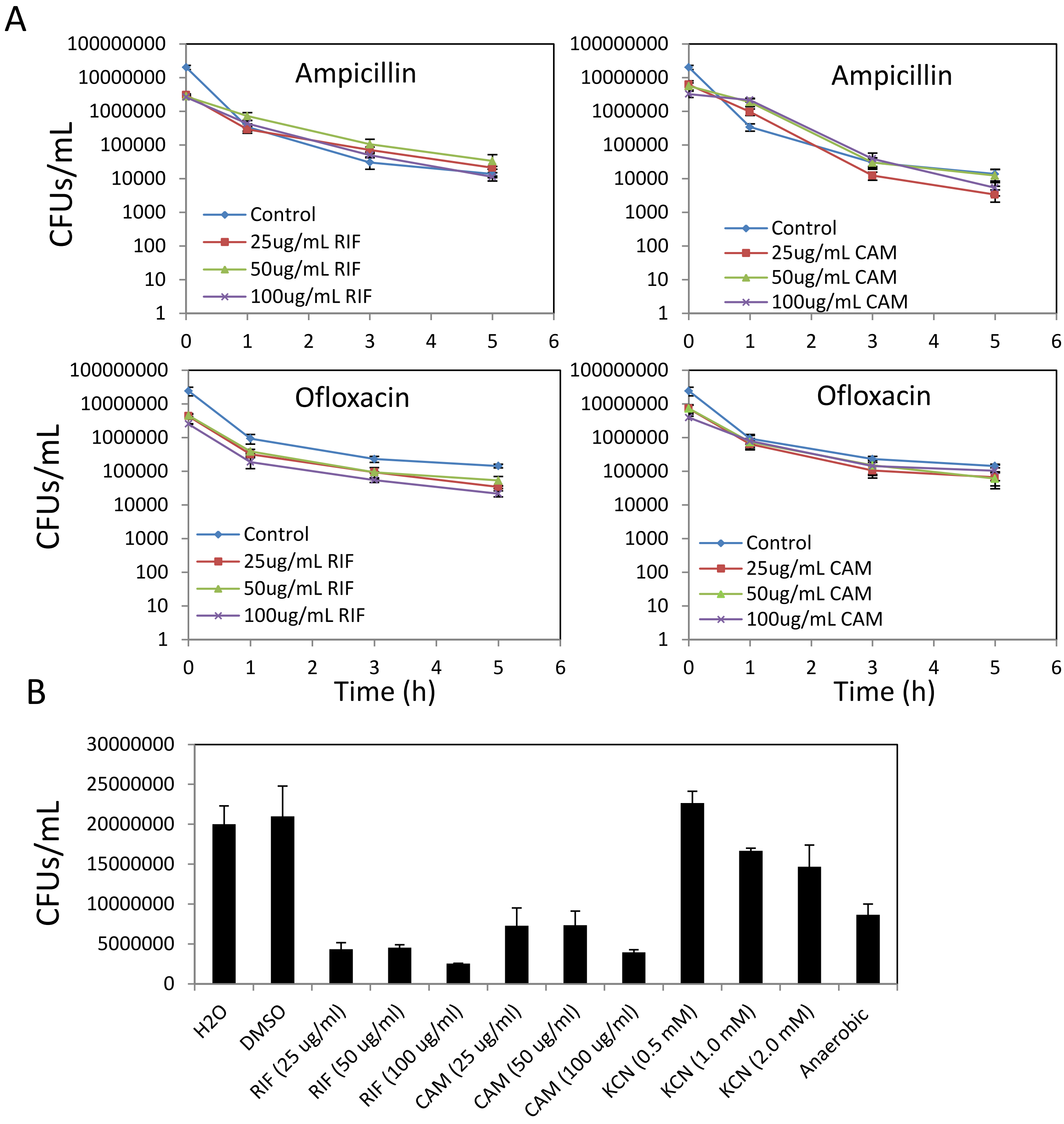

Supplementary Figure 12. Persister levels in RIF and CAM treated cultures.

(A) Overnight cultures were treated with inhibitors at indicated concentrations at early stationary phase (t=6 h), and at t=24 h cells were washed, diluted in fresh LB, and treated with ampicillin or ofloxacin for 5 h to monitor the CFU levels. **(B)** CFUs levels of diluted cultures just before the antibiotic treatment were plotted. Note that this plot is on a linear scale whereas most of the kill-curves are on a log-scale. At least three biological replicates were performed for each experimental condition. Each data point was denoted by mean value ± standard error.

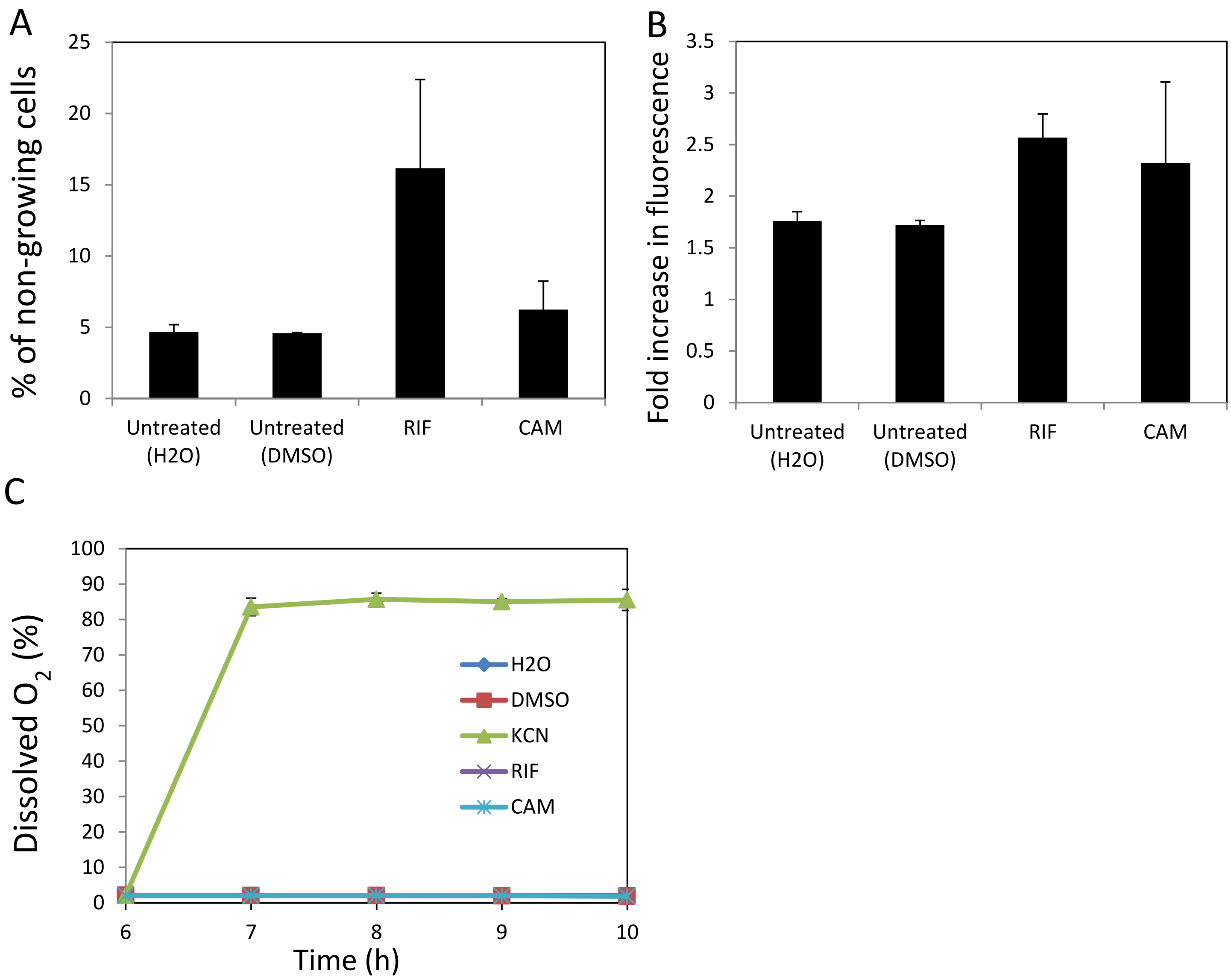

Supplementary Figure 13. Treatment of early stationary phase cells with chemical inhibitors.

Overnight cultures were treated with chemical inhibitors (1 mM KCN, 50 µg/mL RIF and 50 µg/mL CAM) or removed to an anaerobic chamber at early stationary phase (t=6 h), and at t=24 h cells were washed and diluted in fresh LB. When MO001 was used to determine the non-growing cell levels, IPTG was added to overnight cultures. (**A**) Non-growing cells were determined by counting mCherry positive cells at t=2.5 h with a flow cytometer. (**B**) Protein expression capabilities of stationary phase cells carrying pQE-80L*gfp* were performed by inducing *gfp* expression with IPTG for 10 mins after inoculating the cells in fresh media. Fold changes of GFP within 10 min were determined with a flow cytometer. **(C)** Dissolved oxygen concentrations in cultures after treatment with chemicals at t=6h were measured with an oxygen meter. Note that KCN treatment significantly reduced the oxygen utilization compared to control group (H_2O) (p-value<0.05, t-test). At least three biological replicates were performed for each experimental condition. Each data point was denoted by mean value ± standard error

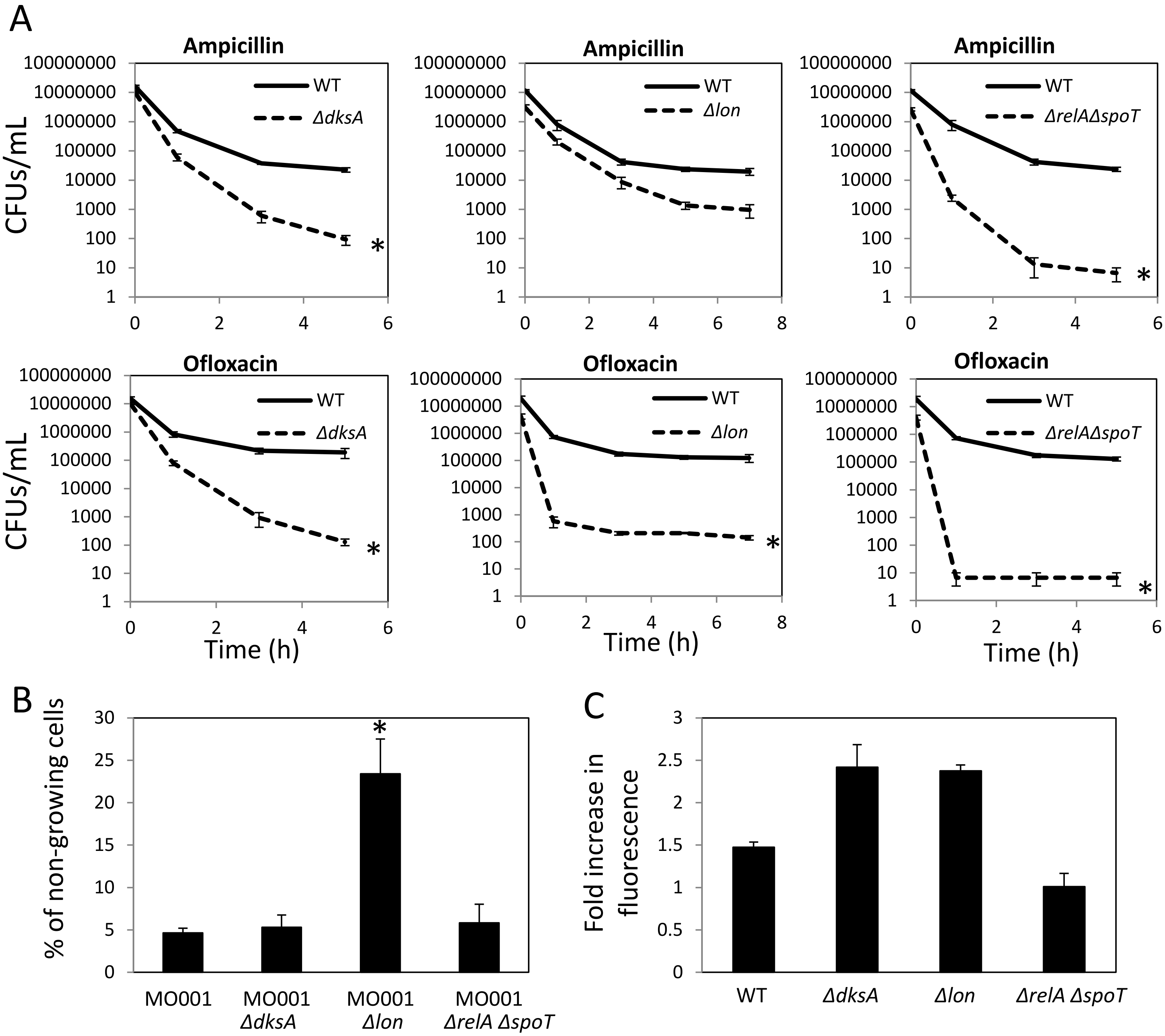

Supplementary Figure 14. Analysis of Δ*dksA*, Δ*lon*, and Δ*relA*Δ*spoT*.
(A) Overnight cultures of Δ*dksA*, Δ*lon* and Δ*relA*Δ*spoT* strains at t=24 h were diluted (100-fold) in fresh LB and treated with ampicillin and ofloxacin. CFU levels were monitored for 5 h during the treatments. **(B)** Overnight cultures of Δ*dksA*, Δ*lon*, and Δ*relA*Δ*spoT* mutants in MO001 background were washed to remove the inducer, diluted in fresh LB, and the non-growing cells were enumerated at t=2.5 h with flow cytometry. **(C)** Overnight cultures of Δ*dksA*, Δ*lon*, and Δ*relA*Δ*spoT* strains carrying *gfp* (without IPTG) were diluted in fresh LB with the inducer, and GFP expression was monitored within 10 min with flow cytometry. * signifies significant differences for comparisons to WT or MO001 (p-value<0.05, t-test). At least three biological replicates were performed for each experimental condition. Each data point was denoted by mean value ± standard error.

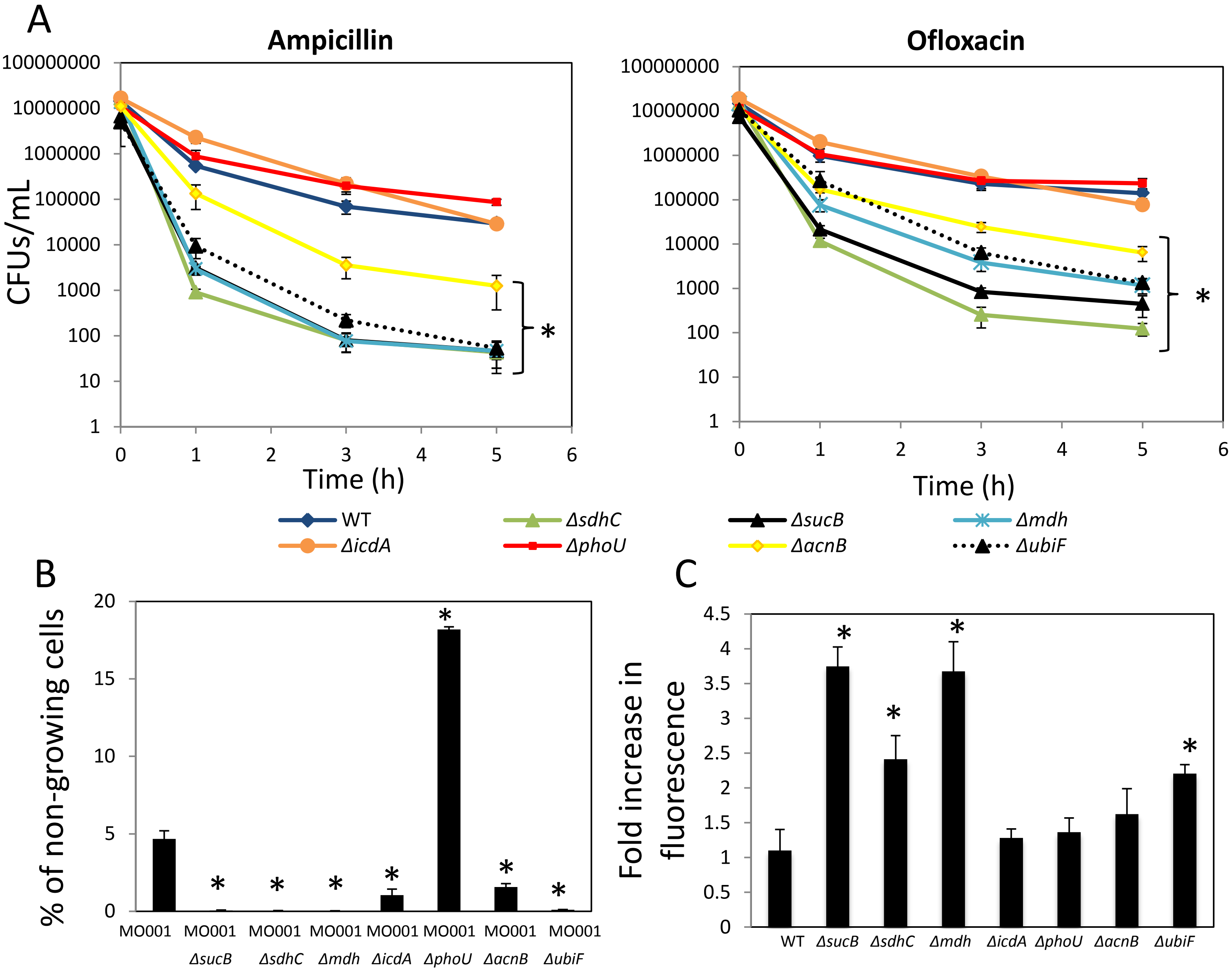

Supplementary Figure 15. Persister levels in metabolic mutants.

(A) Overnight cultures of indicated strains were diluted in fresh LB and treated with ampicillin and ofloxacin to monitor the CFU levels. **(B-C)** Non-growing cell abundance and fluorescence protein expression capabilities of these strains were determined. * signifies significant differences for comparisons to WT or MO001 (p-value<0.05, t-test). At least three biological replicates were performed for each experimental condition. Each data point was denoted by mean value ± standard error.

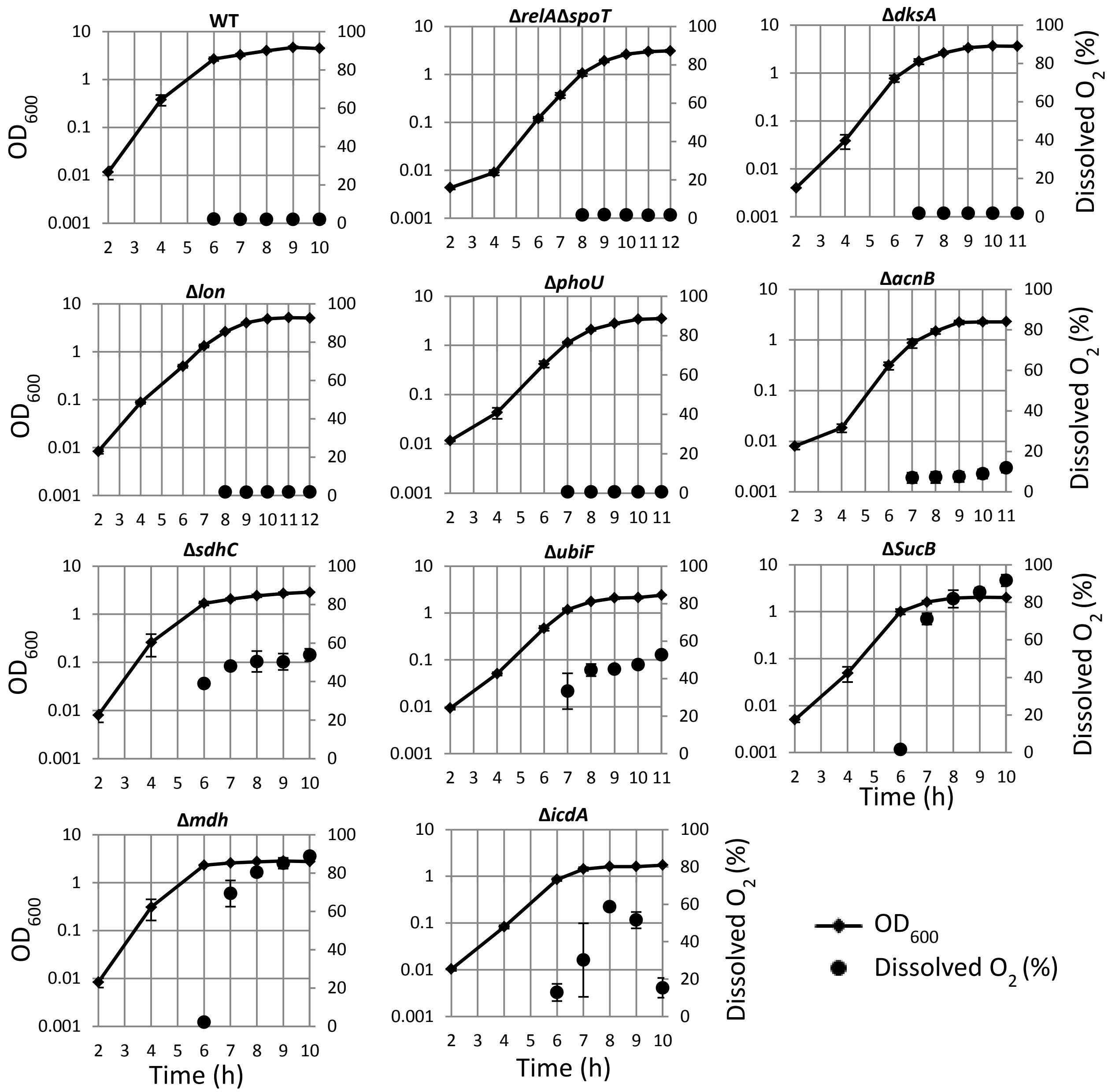

Supplementary Figure 16. Respiratory activity of mutants

Percentages of dissolved oxygen with respect to saturated media were measured in cultures during the early stationary phase. At least three biological replicates were performed for each experimental condition. Each data point was denoted by mean value ± standard error. Note that only Δ*sucB*, Δ*mdh*, Δ*sdhC*, Δ*ubiF* and Δ*icdA* have significantly decreased oxygen utilization at early stationary phase compared to WT (p-value<0.05, t-test); however, we note that the perturbation to respiratory function in Δ*icdA* was transient.

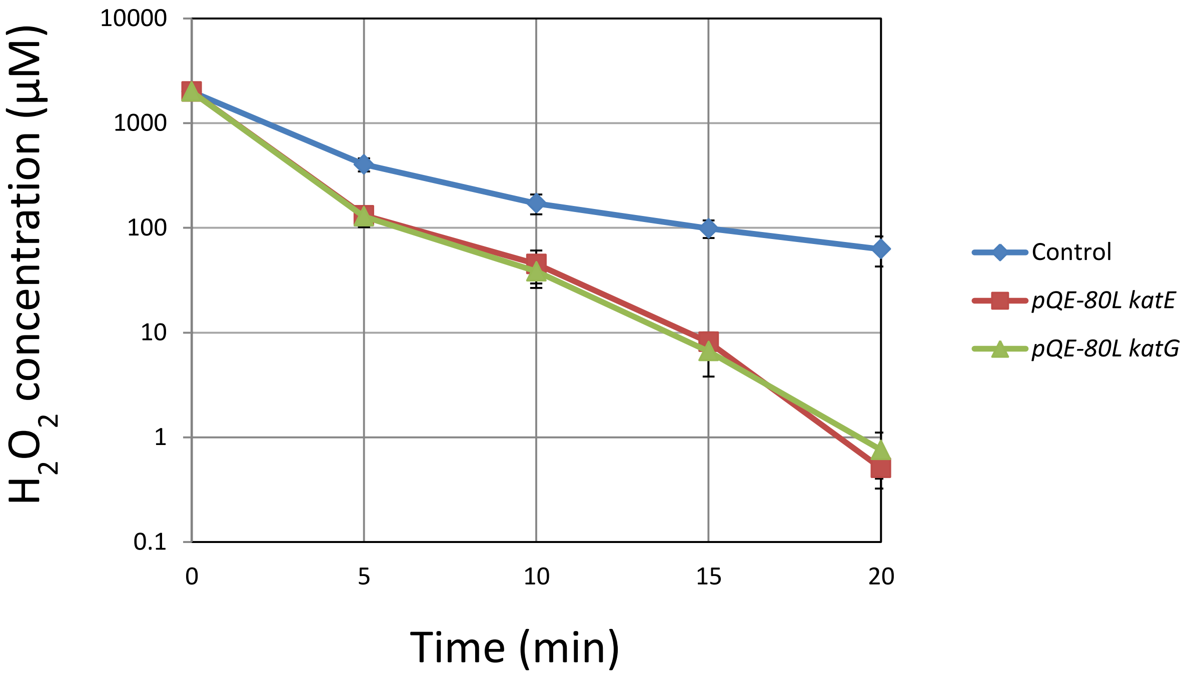

Supplementary Figure 17. H_2O_2 detoxification in strains with over-expressed *katE* or *katG*.

Overnight cultures of *E. coli* carrying empty vector or pQE-80L with *katE* or *katG* were treated with 1mM IPTG to induce protein production at t=6 h. At t=8 h, cells were washed to remove the inducer, and then diluted in M9 salt media to OD_{600}~0.01 in test tubes. The cell cultures were treated with 2000 µM H_2O_2, and incubated at 37 ºC with shaking and H_2O_2 concentrations at indicated time points were measured. At least three biological replicates were performed for each experimental condition. Each data point was denoted by mean value ± standard error. Note that over-expressing KatE or KatG significantly increased H_2O_2 detoxification compared to control group (p-value<0.05, t-test, for time points 5, 10, 15, and 20 min).

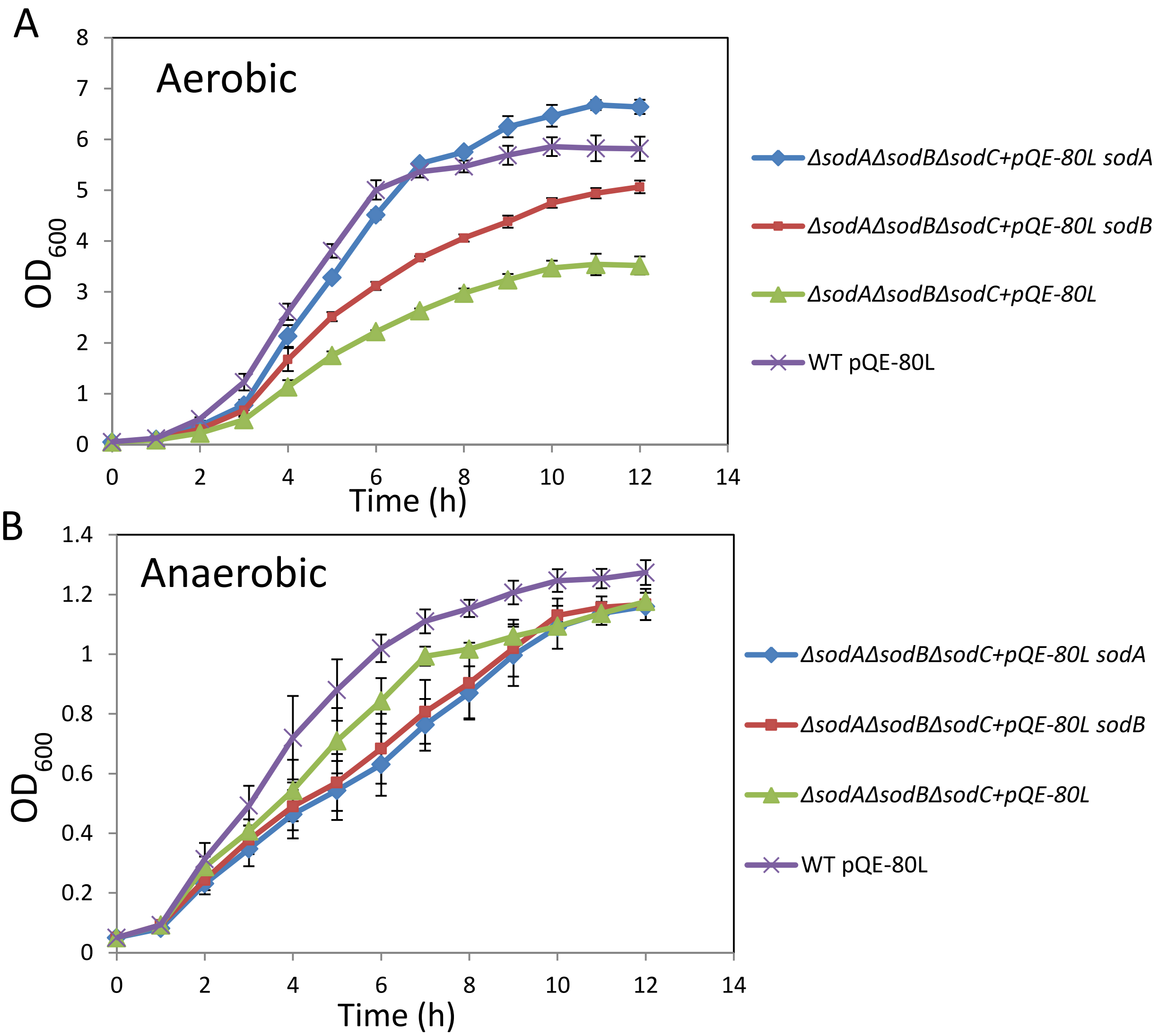

Supplementary Figure 18. Catalytic competency of over-expressed *sodA* and *sodB*.

Overnight cultures of WT and SOD-deficient mutant (Δ*sodA*Δ*sodB*Δ*sodC*) carrying specified plasmids were incubated in 2 mL LB medium with 40 mM $NaNO_3$ in a test tube and cultured at 37 ºC with shaking (250 rpm) for 24 h in an anaerobic chamber. **(A)** Overnight cultures from the anaerobic chamber were diluted to OD_{600}~0.05 in 10 mL LB with 1mM IPTG in 250 mL buffled flasks, and cultured aerobically at 37 ºC with shaking (250 rpm). Note that over-expression of SodA or SodB in the SOD-deficient mutant significantly increased cell growth compared to control group (SOD-deficient mutant with empty vector) under aerobic conditions (p-value<0.05, t-test). **(B)** Overnight cultures from the anaerobic chamber were diluted to OD_{600}~0.05 in 10 mL LB with 1 mM IPTG + 40 mM $NaNO_3$ in 250 mL buffled flasks, and cultured anaerobically at 37 ºC with shaking (250 rpm). Over-expression of SodA or SodB in the SOD-deficient mutant did not significantly improve growth when compared to the empty vector control under anaerobic conditions (p-value>0.05, t-test). At least three biological replicates were performed for each experimental condition. Each data point was denoted by mean value ± standard error.

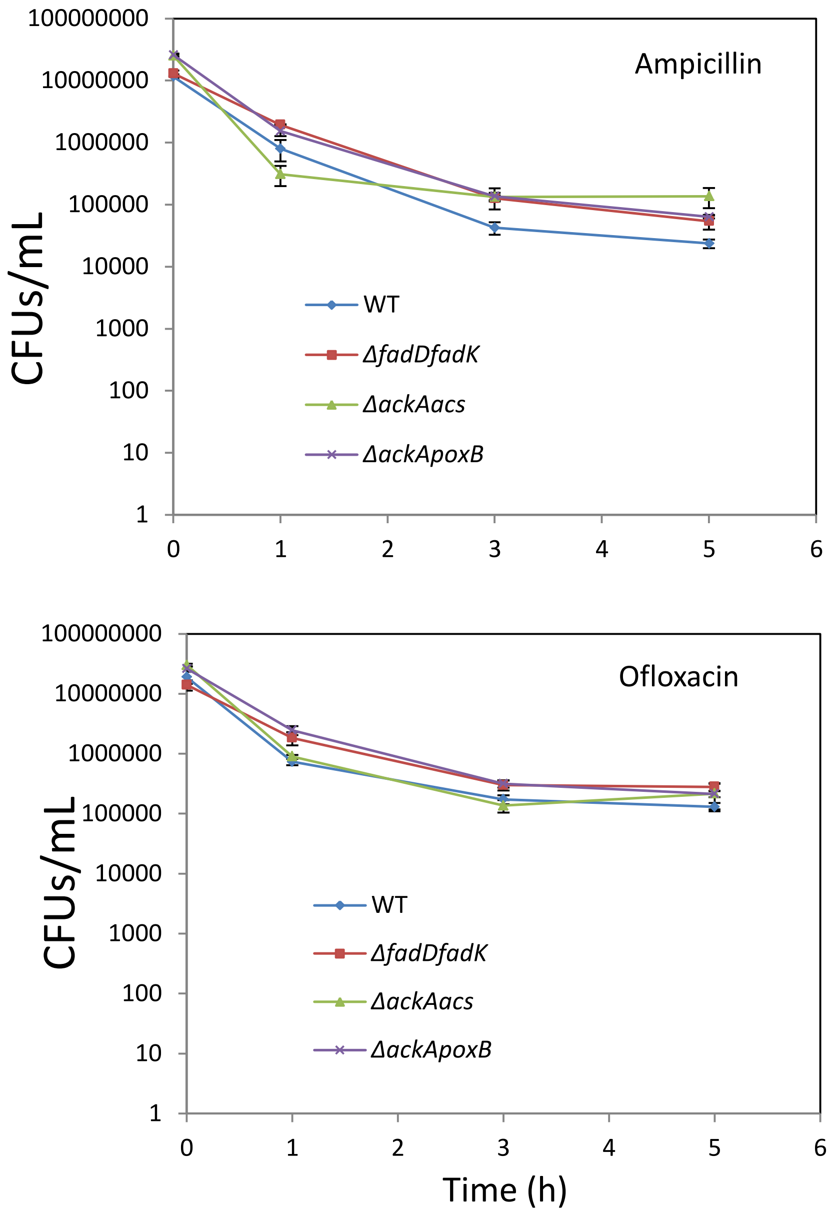

Supplementary Figure 19. Persister levels in Δ*fadD*Δ*fadK*, Δ*ack*Δ*acs*, and Δ*ack*Δ*poxB*.

Stationary phase cells were diluted in fresh LB and treated with ampicillin or ofloxacin for 5 h to monitor the CFU levels. At least three biological replicates were performed for each experimental condition. Each data point was denoted by mean value ± standard error.

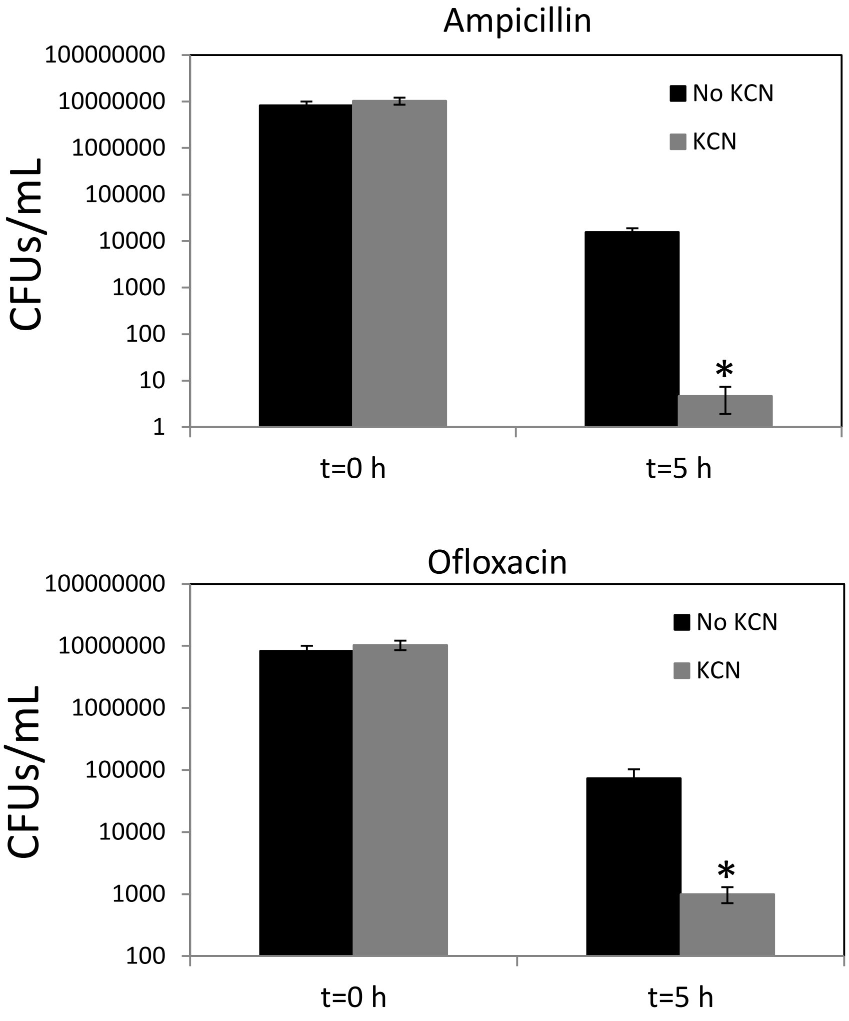

Supplementary Figure 20. Effect of washing step on persister levels of GFP-ssrA expressing samples.

Early stationary phase cultures (t=6 h) where ssrA-tagged *gfp* expression had been induced with 1 mM IPTG were washed to remove inducer and re-suspended in filter-sterilized spent media (without inducer) obtained from WT cultures grown under identical conditions. Cultures were then immediately treated with KCN to inhibit respiration. At t=24 h, cells were washed, diluted in fresh media, and treated with ampicillin or ofloxacin. CFU levels at t=0 h and t=5 h were counted by plating the cell cultures on the agar plates during the treatment. At least three biological replicates were performed for each experimental condition. Each data point was denoted by mean value ± standard error. *Note that KCN treatment significantly reduced persister levels compared to untreated control (p-value<0.05, t-test).

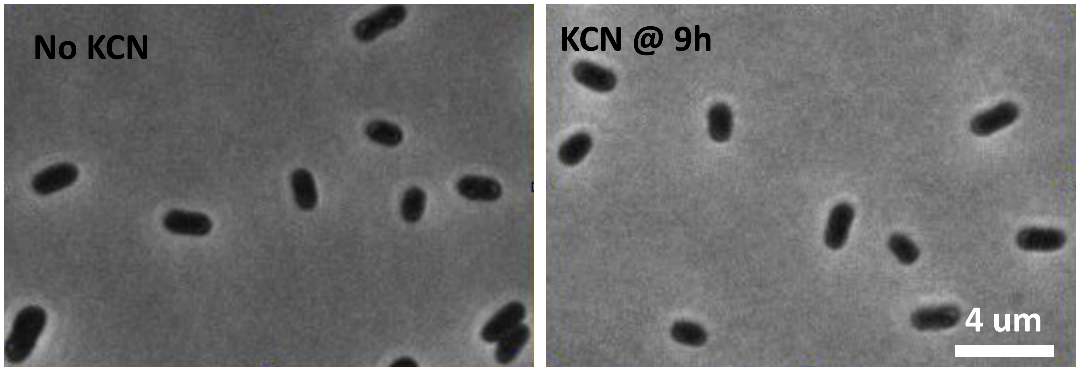

Supplementary Figure 21. Cell size of stationary phase cells treated with KCN at t=9 h.

Stationary phase cells were treated with KCN at t=9 h. At t=24 h, cells were analyzed with a microscope.

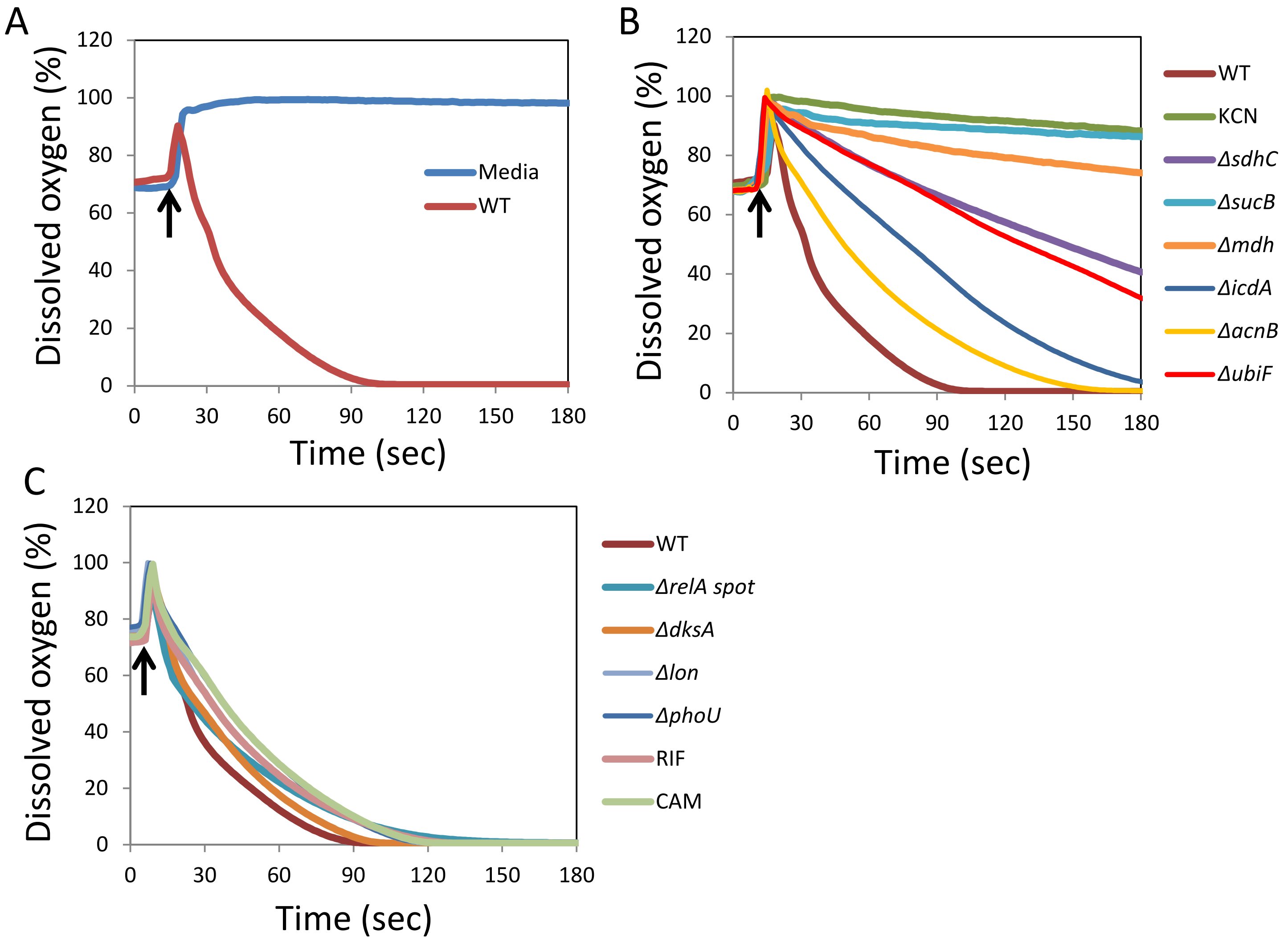

Supplementary Figure 22. Dissolved oxygen measurements.

LB media without cells at 37°C were used to calibrate the oxygen probe. Each data point was represented as a percentage of saturated oxygen concentration obtained from LB media without cells. Arrows indicate the time when the probe was inserted into cultures. **(A)** Percentages of saturated oxygen concentrations in LB media and WT cultures at early stationary phase, **(B)** percentages of saturated oxygen concentrations in WT, KCN-treated WT, Δ*sdhC*, Δ*sucB*, Δ*mdh*, Δ*icdA*, Δ*acnB* and Δ*ubiF* at early stationary phase, and **(C)** percentages of saturated oxygen concentrations in WT, Δ*relA*Δ*spoT*, Δ*dksA*, Δ*lon*, Δ*phoU*, and RIF- and CAM-treated WT cultures at early stationary phase. Dissolved oxygen concentrations reported reflect the concentration measured at 120 seconds. We note that this method provides a relative measure of respiratory activity and not the exact oxygen concentration experienced by the cells during culturing, because these measurements had to be conducted in the absence of shaking.

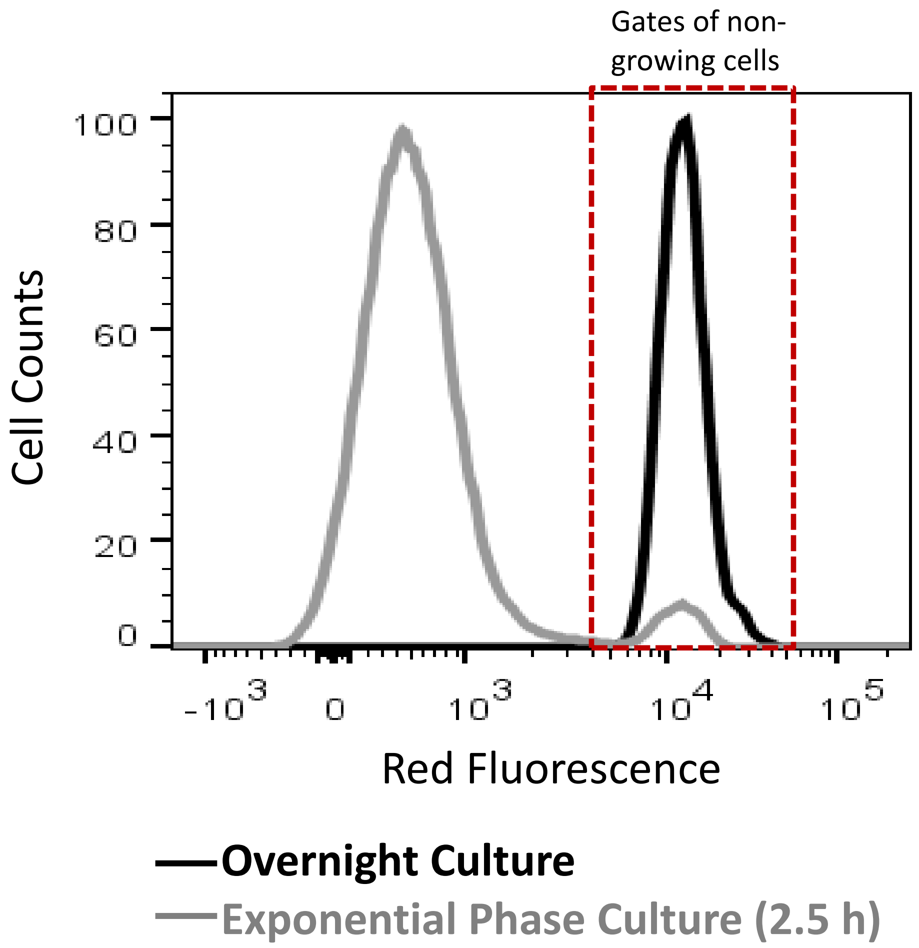

Supplementary Figure 23. Gates of non-growing cell populations.

MO001 cells were cultured with 1mM IPTG to express *mCherry*. After 24 h, cells were washed to remove IPTG, cell pellets were diluted (100-fold) in 1mL of fresh media, and cultured without inducer for 2.5 h at 37 ºC and 250 rpm. Cell cultures at t=0 h and 2.5 h were washed and suspended in 1xPBS and analyzed immediately with a flow cytometer to quantify mCherry levels. As depicted in the figure, all cells started with high red fluorescence (overnight cultures, black line) and as cells divided the red fluorescence of the populations declined, except for small subpopulations in both A and D (non-growing cells) whose fluorescence remained constant due to lack of division (t=2.5 h, gray line). Gates to quantify the abundance of non-growing cells were determined using overnight cultures, and a representative histogram of the gating strategy is provided.

Supplementary Table 1. Primers used for cloning.

Primer Sets	Forward primer (5`---3`)	Reverse primer(5`---3`)	Explanation
1	GCGCATGAATTCATTAAAGAGGAGACCTCTAGTatgAGCAAAGGAGAAGAACT	GCGCCTGGATCCttaTTTGTAGAGCTCATCCATGC	Primers for amplifying *gfp* which was cloned into pQE-80L plasmid. Forward primer has EcoRI cut site and ribosomal binding site (RBS), and reverse primer has BamHI cut site. The gene and plasmid were digested with EcoRI and BamHI before ligation step.
2	GCGCATCAATTGATTAAAGAGGAGACCTCTAGTatgAGCAAAGGAGAAGAACT	GCGCGGAAGCTTttaAGCTGCTAAAGCGTAGTTTTCGTCGTTTGCGACTTTGTAGAGCTCATCCATGC	Primers used to introduce *ssrA* tag (reverse primer) to *gfp*.
3	GCGCATCAATTGATTAAAGAGGAGACCTCTAGTatgAGCAAAGGAGAAGAACT	GCGCGGGGATCCttaAGCTGCTAAAGCGTAGTTTTCGT	Primers for amplifying *gfp-ssrA*, which was cloned into pQE-80L plasmid. Forward primer has MfeI cut site and RBS, and reverse primer has BamHI cut site. The gene was digested with MfeI and BamHI, and plasmid was digested with EcoRI and BamHI before ligation step.
4	GCGCATGGTACCCCGGGTCGACCTGCAGCCA	GCGCCTGAGCTCGAATTCTGTGTGAAATTGTTATCCG	Primers for amplifying entire pQE-80L plasmid to introduce KpnI and SucI cut sites. Forward primer has KpnI cut site, and reverse primer has SacI cut site. These primers were used when the insert gene had EcoRI and MfeI cut sites.
5	GCGCATGAGCTCATTAAAGAGGAGACCTCTAGTatgGTGAGCAAGGGCGAGGAGGATA	GCGCCTGGTACCAGTctaCTTGTACAGCTCGTCCATGCCG	Primers for amplifying *mCherry*, which was cloned into pQE-80L plasmid. Forward primer has SacI cut site and RBS, and reverse primer has KpnI cut site. The plasmid was amplified with primer set 4. Both gene and plasmids were digested with SacI and KpnI before ligation step.
6	GCGCATGAATTCATTAAAGAGGAGACCTCTAGTatgAGCTATACCCTGCCATCCCTGC	GCGCCTGGATCCAGTttaTTTTTTCGCCGCAAAACGTGCC	Primers for amplifying *sodA*, which was cloned into pQE-80L plasmid. Forward primer has EcoRI cut site and RBS, and reverse primer has BamHI cut site. The gene and plasmid were digested with EcoRI and BamHI before ligation step.
7	GCGCATCAATTGATTAAAGAGGAGACCTCTAGTatgTCATTCGAATTACCTGCACTAC	GCGCCTGGATCCAGTttaTGCAGCGAGATTTTTCGCTACG	Primers for amplifying *sodB, which* was cloned into pQE-80L plasmid. Forward primer has MfeI cut site and RBS, and reverse primer has BamHI cut site. The gene was digested with MfeI and BamHI, and plasmid was digested with EcoRI and BamHI before ligation step.
8	GCGCATCAATTGATTAAAGAGGAGACCTCTAGTatgTCGCAACATAACGAAAAGAACC	GCGCCTGAGCTCAGTtcaGGCAGGAATTTTGTCAATCTTA	Primers for amplifying *katE*, which was cloned into pQE-80L plasmid. Forward primer has MfeI cut site and RBS, and reverse primer has BamHI cut site. The gene was digested with MfeI and BamHI, and plasmid was digested with EcoRI and BamHI before ligation step.
9	GCGCATGAGCTCATTAAAGAGGAGACCTCTAGTatgAGCACGTCAGACGATATCCATA	GCGCCTGGTACCAGTttaCAGCAGGTCGAAACGGTCGAGG	Primers for amplifying *katG*, which was cloned into pQE-80L plasmid. Forward primer has SacI cut site and RBS, and reverse primer has KpnI cut site. The plasmid was amplified with primer set 4. Both gene and plasmids were digested with SacI and KpnI before ligation step.
